# Integration of DNA methylation patterns and genetic variation in human pediatric tissues help inform EWAS design and interpretation

**DOI:** 10.1186/s13072-018-0245-6

**Published:** 2019-01-02

**Authors:** Sumaiya A. Islam, Sarah J. Goodman, Julia L. MacIsaac, Jelena Obradović, Ronald G. Barr, W. Thomas Boyce, Michael S. Kobor

**Affiliations:** 1 Department of Medical Genetics, University of British Columbia, Vancouver, BC Canada; 2 Centre for Molecular Medicine and Therapeutics, BC Children’s Hospital, Vancouver, BC Canada; 3 Graduate School of Education, Stanford University, Palo Alto, CA USA; 4 Department of Pediatrics, University of British Columbia, Vancouver, BC Canada; 5 Departments of Pediatrics and Psychiatry, University of California, San Francisco, CA USA; 6 Human Early Learning Partnership, University of British Columbia, Vancouver, BC Canada; 7 Canadian Institute for Advanced Research, Toronto, ON Canada

**Keywords:** DNA methylation, Genetic variation, Surrogate tissues, Peripheral blood leukocytes, Buccal epithelial cells, Illumina 450K array, Pediatric

## Abstract

**Background:**

The widespread use of accessible peripheral tissues for epigenetic analyses has prompted increasing interest in the study of tissue-specific DNA methylation (DNAm) variation in human populations. To date, characterizations of inter-individual DNAm variability and DNAm concordance across tissues have been largely performed in adult tissues and therefore are limited in their relevance to DNAm profiles from pediatric samples. Given that DNAm patterns in early life undergo rapid changes and have been linked to a wide range of health outcomes and environmental exposures, direct investigations of tissue-specific DNAm variation in pediatric samples may help inform the design and interpretation of DNAm analyses from early life cohorts. In this study, we present a systematic comparison of genome-wide DNAm patterns between matched pediatric buccal epithelial cells (BECs) and peripheral blood mononuclear cells (PBMCs), two of the most widely used peripheral tissues in human epigenetic studies. Specifically, we assessed DNAm variability, cross-tissue DNAm concordance and genetic determinants of DNAm across two independent early life cohorts encompassing different ages.

**Results:**

BECs had greater inter-individual DNAm variability compared to PBMCs and highly the variable CpGs are more likely to be positively correlated between the matched tissues compared to less variable CpGs. These sites were enriched for CpGs under genetic influence, suggesting that a substantial proportion of DNAm covariation between tissues can be attributed to genetic variation. Finally, we demonstrated the relevance of our findings to human epigenetic studies by categorizing CpGs from published DNAm association studies of pediatric BECs and peripheral blood.

**Conclusions:**

Taken together, our results highlight a number of important considerations and practical implications in the design and interpretation of EWAS analyses performed in pediatric peripheral tissues.

**Electronic supplementary material:**

The online version of this article (10.1186/s13072-018-0245-6) contains supplementary material, which is available to authorized users.

## Background

Epigenome-wide association studies (EWASs) are becoming increasingly popular, in part due to their potential to enhance our understanding of the determinants of health and disease, including potential early life embedding of experiences and exposures and their association with later life outcomes [1–7]. The term “epigenetics” describes mitotically heritable modifications of DNA and its regulatory components, including chromatin and non-coding RNA, that potentially modulate cellular states or fate through gene expression changes, without changing the DNA sequence itself [8–10]. DNA methylation (DNAm), which involves the covalent attachment of a methyl group to a cytosine primarily at cytosine–phosphate–guanine (CpG) dinucleotides, is the most well-studied chromatin mark in human populations due to its relative stability and ease of measurement on quantitative array-based methods [11, 12]. To date, EWASs have identified differential DNAm across a broad range of contexts including disease states, genetic background and environmental exposures, thereby providing evidence for the potential contribution of DNAm in mediating gene-by-environment ((G × E)) interactions [1, 13, 14].

Given that tissue specificity is an integral feature of epigenetic profiles, as different tissues and cell types acquire distinct epigenomes during differentiation, the selection of tissue source is a key consideration in the careful design and interpretation of EWAS analyses [15–17]. The collection of a disease-relevant, target tissue allows for the direct assessment of epigenetic associations that may be implicated in the underlying phenotypic or disease biology. In certain cases, readily accessible peripheral tissues may represent the target tissue; for example, use of PBMCs for the investigation of DNAm associations to immune or inflammatory phenotypes [4, 18–20]. However, in many cases, the target tissue, such as brain, muscle, adipose tissue, among others, may be impossible or very difficult to collect from living individuals or at sufficient quality for analysis from postmortem samples [3]. Easily accessible peripheral tissues are therefore often used in human epigenetic studies for biomarker discovery in lieu of target tissues that are difficult to collect. This is particularly relevant to pediatric cohorts in which biopsy specimens with invasive collection procedures or postmortem samples are less common than in adult populations. As such, more readily accessible tissues with minimally invasive collection procedures, such as cord blood, saliva, buccal epithelium cells (BECs) or peripheral blood mononuclear cells (PBMCs), are widely used tissue source materials for early life EWASs. The use of pediatric tissues in DNAm analyses is further complicated by the fact that widespread alterations occur in tissue-specific DNAm patterns during development, therefore conferring additional complexity in the selection of appropriate source material for early life DNAm studies [21, 22]. Furthermore, changes in cell composition within a tissue are a source of potential confound in EWAS, as shown for a number of DNAm associations, including changes during development and certain environmental exposures such as smoking [1, 23–27].

Currently, two major focal points in human epigenetic research are to elucidate the tissue specificity of DNAm patterns with respect to individual CpGs as well as assess inter-individual variation within a single tissue [21, 28–30]. At a population level, a number of studies have examined the concordance of DNAm patterns across multiple tissues [20, 29–34]. Findings have shown that beyond tissue-specific differences in absolute DNAm measures, inter-individual DNAm variability also varies by tissue type [20, 31]. For example, previous work by our group has shown that BECs have greater DNAm variability over matched PBMCs at both the genome-wide level and at individual CpGs [20]. Moreover, CpG sites with higher DNAm variability tend to be more correlated between matched tissues [29–31, 34]. Although these results provide important insights into the comparability of DNAm measures across matched tissues, the analyses to date have been conducted in adult tissues, thereby limiting their relevance to DNAm profiles from pediatric samples. As previous studies have demonstrated that developmental changes in blood DNAm patterns tend to be more pronounced and occur more rapidly in childhood, the examination of DNAm concordance and variability in pediatric tissues represents an important and currently missing step in our understanding of EWAS associations from pediatric peripheral tissues [21, 22].

Genetic variation represents an additional contributor to DNAm patterns in tissues, with genetic influences accounting for nearly 20–80% of DNAm variance within a tissue [35–40]. Methylation quantitative trait loci (mQTL), sites at which DNAm is associated with genetic variation, are present across the genome and are often consistent across tissues, ancestral populations and developmental stage [41–44]. Notably, genetically influenced sites of inter-individual DNAm variation, which can co-occur across tissues, may be biologically informative. For example, allele-specific DNAm of the FK605 binding protein 5 (*FKBP5*) gene, which has been associated with risk of developing stress-related psychiatric disorders, responds to glucocorticoid stimulation in a similar way in peripheral blood cells and neuronal progenitor cells [45]. Within a particular tissue, such as blood, mQTL often are stable across development [43, 46]. Moreover, approximately 75% of the inter-individual regional DNAm variance within a single tissue can be best described by (G × E) models [47]. As such, delineating the contribution of genetic influences to tissue-specific DNAm may help clarify the interpretation of EWAS associations.

Given that early life development brings about sizable changes to DNAm patterns, it is important to examine DNAm variability and concordance between peripheral tissues, as well as genetic influences on early life DNAm patterns, in childhood [21, 22]. To this end, we used matched PBMC and BEC samples, two commonly used peripheral tissues in EWAS, from two independent early life cohorts in order to identify (a) differences in inter-individual variability and concordance of DNAm between these tissues and (b) genetic contributions to these patterns at the site-specific level. Our results showed that genome-wide DNAm variability differed between tissues, with BECs exhibiting greater inter-individual DNAm variability over PBMCs. Moreover, we found that highly variable CpGs were more likely to be positively correlated between matched tissues and enriched for DNAm sites under genetic influence. Finally, we demonstrated the relevance of our findings to EWAS analysis by categorizing DNAm associations that were previously identified in pediatric BECs and peripheral blood. Collectively, these findings highlighted a number of potential insights and considerations for the appropriate design and interpretation of EWAS analyses performed in commonly used peripheral tissues of pediatric samples.

## Results

### Study cohorts and DNAm data processing

To explore the tissue-specific DNAm patterns of pediatric PBMCs and BECs, we used subsets from two independent human cohorts, GECKO and C3ARE, both of which contained matched tissue samples from healthy children from the Lower Mainland Vancouver area. In GECKO, individuals ranged in age from 6 to 11 years at time of BEC collection (median = 8.8) and 7 to 13 years at time of PBMC collection (median = 10.3). Of the GECKO study sample (*n* = 79), 46% were female (*n* = 36). In C3ARE (*n* = 16), individuals ranged in age from 3 to 5 years at time of BEC collection (media*n* = 4.5) and 4 to 5 years at time of PBMC collection (median = 5.1) and 50% were female (*n* = 8) (Table 1).

**Table 1:** Sample characteristics for C3ARE and GECKO cohorts

Characteristics	C3ARE	GECKO
Age range (years) at BEC collection (mean)	3.7–5.8 (4.5)	6–11 (8.8)
Age range (years) at PBMC collection (mean)	4.2–5.9 (5.1)	7–13 (10.3)
Sex	*n* = 16 total (50% F)	*n* = 79 total (46% F)

DNAm data, as measured across ~ 485,000 CpGs by the Illumina 450K array, were filtered down to overlapping 419,507 sites which passed independent quality control measures in both cohorts. Each 450K dataset was normalized to remove probe type differences and adjusted for cell type heterogeneity in each tissue using established bioinformatic correction methods [34, 48–51]. Genetic variants were measured genome-wide using the Illumina Infinium PsychChip. Following probe filtering for low-quality probes, 550,200 and 547,662 SNP probes remained for analysis in C3ARE and GECKO, respectively. We used these corrected DNAm and genotyping data of matched PBMC and BEC samples from both cohorts to assess inter-individual DNAm variability, DNAm concordance across tissues and genetic influence on DNAm, in order to gain insight into DNAm variation in these commonly used pediatric peripheral tissues.

### BEC DNAm had significantly greater inter-individual variability than PBMC DNAm

**Fig. 1:**
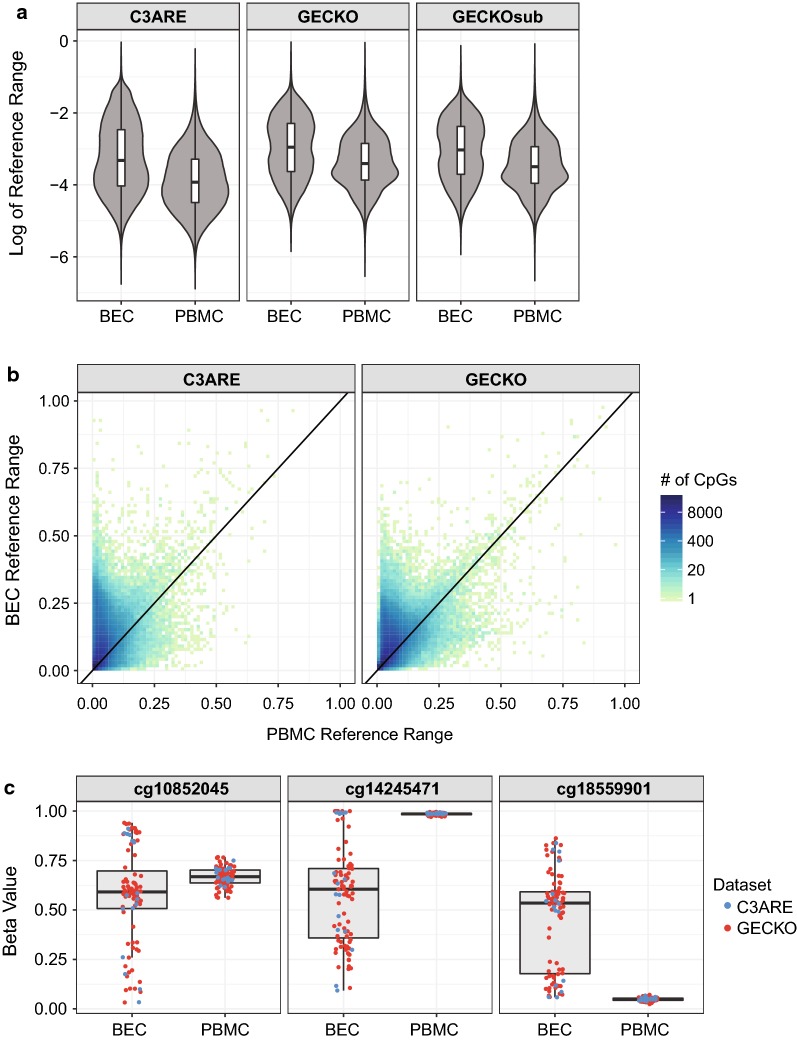
BEC DNAm was consistently more variable than PBMC DNAm at the genome-wide and probewise level. **a** Distribution of reference range in C3ARE, GECKO and GECKOsub, showing significantly great variability in BEC versus PBMC (Wilcoxon *p* < 2.2 × 10^−16^ in each cohort). **b** Scatterplot of PBMC versus BEC reference range in each cohort. **c** Three examples of CpGs with the greatest reference range difference between tissues. Individuals from the GECKO cohort are shown in red and individuals from C3ARE are shown in blue

As inter-individual DNAm variability within a tissue likely relates to the potential effect sizes that are detectable in EWAS analyses, we were interested in assessing tissue-specific DNAm variability. To this end, we first interrogated the global differences in inter-individual DNAm variability between PBMC and BEC samples, following in silico correction for cell type differences in each tissue. We used reference range as a measure of DNAm variability as opposed to absolute range in order to minimize potential skewing by outlier values and non-normal DNAm values at individual CpGs, as previously described [31, 52]. Within each cohort, BEC DNAm had a significantly greater reference range than PBMC DNAm (Fig. 1a; Wilcoxon signed-rank test, all *p* values = 2.2 × 10^−16^). In GECKO, the median reference range, measured in beta values, was 1.9% higher in BECs (5.2%) than in PBMCs (3.3%). Similarly, in C3ARE, the median reference range was 1.6% higher in BECs (3.6%) than in PBMCs (2.0%). The difference in reference range was not dependent on sample size, as demonstrated by the consistency between GECKO and GECKOsub, the GECKO cohort randomly subsampled to the sample size of C3ARE (*n* = 16) 100 times (Fig. 1a). In addition, tissue-specific differences in DNAm variability were observed at individual CpGs, as determined by a Fligner–Killeen test, a nonparametric test measuring homogeneity of variances between two groups. In GECKO, 217,091 probes had significantly greater variability in BEC at FDR ≤ 0.05, while only 32,350 probes were more variable in PBMC. Similarly, in the C3ARE cohort, 127,472 probes had greater variability in BECs (FDR ≤ 0.05) and 8183 probes in PBMCs (FDR ≤ 0.05; Fig. 1b). This consistent difference in variability between BECs and PBMCs was best represented by cg10852045, cg14245471 and cg1855901 (Fig. 1c). Collectively, 85% of C3ARE probes (108,498) with greater variability in BEC were also found in the GECKO cohort to have greater BEC variability. These 108,498 CpGs were enriched for sites with high inter-individual BEC variability in both cohorts (10,000 permutations, *p* value < 1 × 10^−4^). As well, 84% of C3ARE probes (6840) with greater variability in PBMCs, were also more variable in PBMCs in the GECKO cohort; similarly, this subset was enriched for CpGs with high PBMC variability in both cohorts (10,000 permutations, *p* value < 1 × 10^−4^). These findings suggested that BEC DNAm was consistently more variable than PBMC DNAm across both cohorts, in line with previous analyses using adult tissues [20].

Apart from tissue-specific differences in reference range, we also observed a cohort-specific difference in DNAm variability. Specifically, CpGs in GECKO had a significantly greater median reference range than C3ARE CpGs in both tissues (Wilcoxon rank-sum test, *p* values = 2.2 × 10^−16^). In BECs, the median reference range was 1.6% higher in GECKO than C3ARE and in PBMCs, it was greater by 1.3%. This difference remained significant when GECKOsub was used in lieu of GECKO (BEC difference = 1.2%, PBMC difference = 1.1%, Wilcoxon rank-sum test, *p* values = 2.2 × 10^−16^), suggesting that these cohort-specific DNAm variability differences occurred irrespective of sample size and may be related to age-associated increases in DNAm variability, as previously described [53–58].

### Variable CpGs were more highly correlated between tissues

**Fig. 2:**
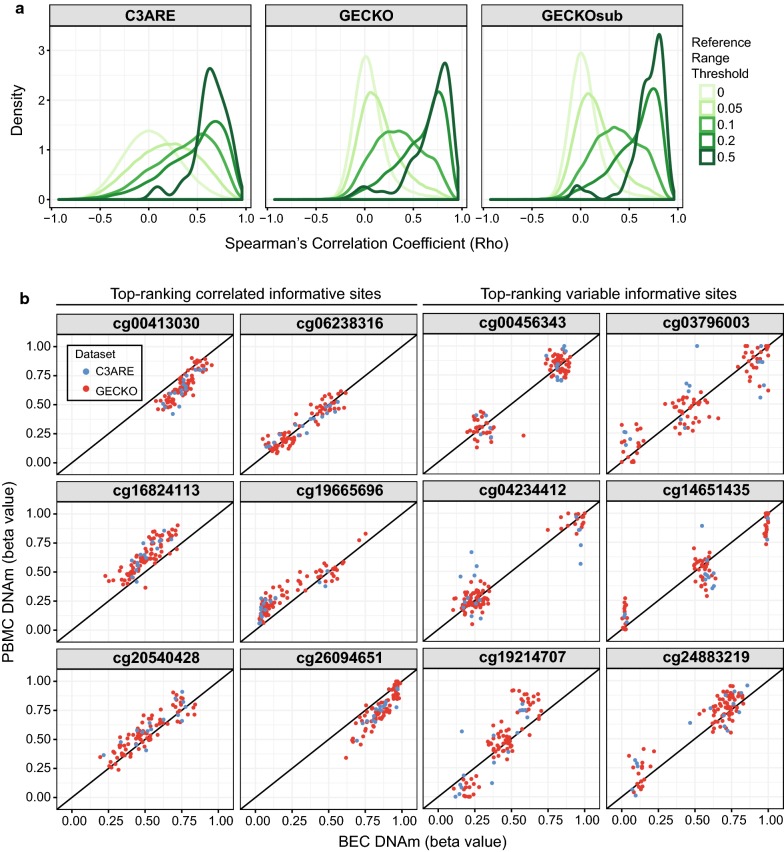
Variable CpGs were more highly correlated between tissues. **a** Density distribution plots of Spearman’s correlation rho between matched PBMCs and BECs across C3ARE, GECKO and GECKOsub datasets showing progressively greater enrichment of highly positively correlated CpGs at increasing reference range thresholds. Reference range thresholds were set along a sliding scale with cut-offs at 0, 0.05, 0.1, 0.2 and 0.5 (depicted by gradient of green lines). **b** Scatterplots of BEC DNAm versus PBMC DNAm for a representative set of informative sites (defined as CpGs that are both variable across individuals and highly correlated between BECs and PBMCs). Top-ranking correlated informative sites (shown in the left two columns) exhibited continuous distributions. In contrast, top-ranking variable informative sites (shown in the right two columns) exhibited discrete distributions, suggesting that these Cps may be under genetic influence. C3ARE samples are shown in blue while GECKO samples are shown in red

Taking advantage of the matched tissue design of our cohorts, we evaluated whether DNAm variation in one tissue reflected DNAm variation in the other. We performed probewise Spearman’s correlations between paired BEC and PBMC samples for the C3ARE, GECKO and GECKOsub datasets, respectively (Additional file 1: Fig. S5). Using multiple reference range thresholds to capture increasingly variable CpGs, as previously described, we observed progressively greater enrichment of highly positively correlated CpGs, irrespective of sample size (Fig. 2a and Additional file 2: Table S1) [31]. This suggested that, broadly speaking, CpGs with greater variability were more likely to be correlated between these tissues than less variable CpGs.

We next sought to investigate DNAm variability and concordance at individual CpGs. Specifically, we aimed to identify “informative sites,” which we defined as CpGs that are both variable across individuals and highly correlated between BECs and PBMCs, using a previously described method [31]. Such CpGs may be predictive of PBMC DNAm when measured in BECs or vice versa. To be classified as informative, i.e., variable and concordant, a CpG was required to have a reference range ≥ 5% in both tissues and meet the minimum correlation coefficient between tissues of 0.47 in GECKO samples and 0.32 in C3ARE samples, as determined by a beta mixture model run on highly variable CpGs in each cohort. Overlapping CpGs that met these criteria in both cohorts resulted in a set of 8140 informative sites. Of note, we observed a greater than expected by chance overlap (3682 out of 8140 sites, 45%, 10,000 permutations, *p* < 1 × 10^−4^) between our set of informative sites and informative CpGs previously identified between matched samples from adult brain and blood tissues [31]. Visualization of our six most correlated informative sites revealed continuous distributions of positively correlated DNAm values between the tissues, as expected (Fig. 2b). However, the most variable informative sites exhibited discrete distributions with 2–3 distinct clusters, rather than a typical continuous distribution, suggesting that these CpGs may be enriched for CpGs which are likely under genetic influence (Fig. 2b) [30].

### Genetic variation contributed to tissue concordance

**Fig. 3:**
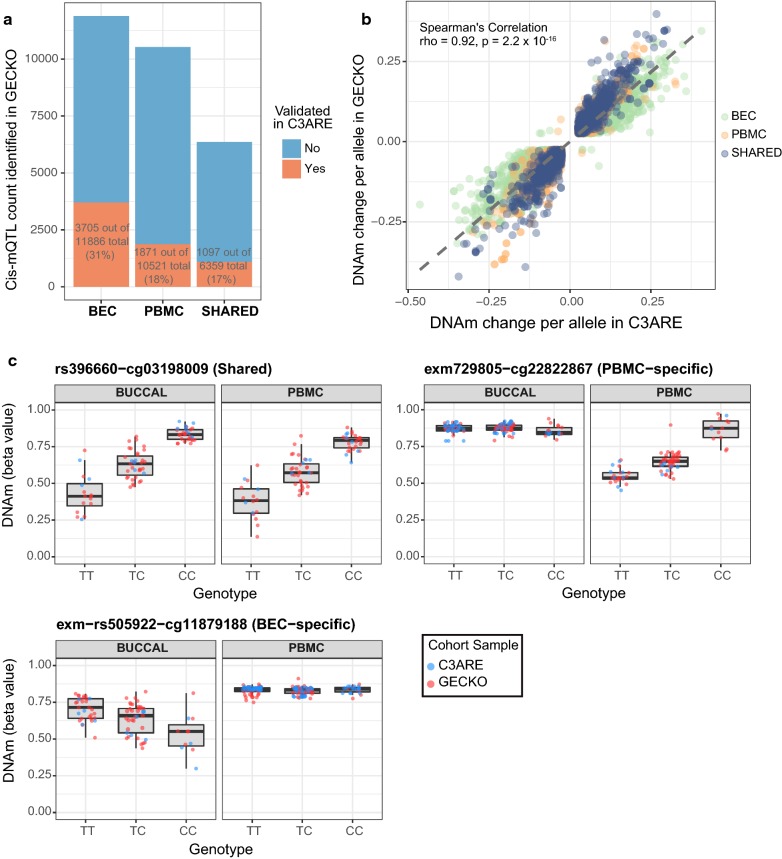
Independently validated *cis*-mQTL were more likely to be shared across tissues than expected by chance. **a** Stacked bar plot representing number of *cis*-mQTL identified in GECKO discovery cohort (shown in blue) and number of *cis*-mQTL validated in C3ARE cohort (shown in red) in either BECs, PBMCs or shared across both tissues. **b** Scatterplot of DNAm change per allele in GECKO versus C3ARE across all validated *cis*-mQTL shows mQTL effect sizes (measured as DNAm change per allele) were highly consistent across cohorts (BEC-specific, PBMC-specific and shared-tissue mQTL shown in different colors). **c** Boxplots of genotype versus DNAm for representative examples of a shared-tissued (top left), PBMC-specific (top right) and a BEC-specific (bottom) validated *cis*-mQTL. C3ARE samples are shown in blue while GECKO samples are shown in red

In order to determine the influence of local genetic variation on inter-individual DNAm variability and concordance of DNAm signal across matched peripheral tissues, we identified *cis*-mQTL in both BEC and PBMC samples, respectively. Briefly, CpGs were filtered by DNAm variability (reference range ≥ 0.05) in their respective tissues and were correlated against all SNPs within a 5 kb window, a window size previously demonstrated to enrich for mQTLs that are more likely to be functionally linked to proximal CpGs [47, 59, 60]. As the GECKO cohort had a larger sample size as compared to C3ARE and was therefore more adequately powered for *cis*-mQTL detection, the GECKO samples were used as the discovery cohort. A total of 16,880 and 18,245 significant *cis*-mQTL were identified in GECKO PBMCs and BECs, respectively (FDR ≤ 0.05 and DNAm change per allele ≥ 2.5%), with 6359 mQTL in common between tissues (Fig. 3a). These mQTLs were selected for validation testing in C3ARE.

After quality control processing and variability filtering of the C3ARE DNAm and genotyping data, 16,138 and 17,563 SNP-CpG pairs could be tested for validation in PBMCs and BECs, respectively (mQTL that were not tested for validation lacked genetic variability in the C3ARE cohort). This resulted in a total of 1871 PBMC-specific, 3705 BEC-specific and 1097 shared-tissue validated *cis*-mQTL (FDR ≤ 0.05 and DNAm change per allele ≥ 2.5%), which exhibited highly consistent effect sizes between GECKO and C3ARE cohorts (Spearman rho = 0.92, *p* = 2.2 × 10^−16^) (Fig. 3a, b). The overlap between validated *cis*-mQTL between tissues was greater than expected by chance (10,000 permutations, *p* value < 1 × 10^−4^) (Fig. 3a and Additional file 3: Fig. S6). This suggested that genetic influences contributed to covariation between tissues. Finally, we found a significant overlap of our 1871 PBMC-specific and 1097 shared-tissue *cis*-mQTL with previously published mQTL hits from whole blood samples of 7-year-old children in the AIRES cohort (1810 out of 2968 sites, 61%, 10,000 permutations, *p* < 1 × 10^−4^), further supporting our mQTL findings [46].

We next sought to characterize our validated *cis*-mQTL by their genomic localization and functional features. Firstly, the 4980 unique CpGs associated with the validated *cis*-mQTL showed a greater than expected by chance enrichment in intergenic regions and were depleted in intragenic and north shelf regions (2–4 kb upstream of CpG islands) (Additional file 4: Fig. S7A, FDR ≤ 0.05). In particular, both the CpGs associated with tissue-specific *cis*-mQTL and the CpGs associated with shared-tissue *cis*-mQTL were significantly enriched at intergenic and intragenic regions and showed significant depletion at promoters and CpG islands, where DNAm levels tend to be low and there is limited inter-individual variation (Additional file 4: Fig. S7B and C, FDR ≤ 0.05). However, tissue-specific mQTL CpGs exhibited significant enrichment at south shelf regions (2–4 kb downstream of CpG islands), whereas shared-tissue mQTL CpGs were significantly enriched in north shores (0–2 kb upstream of CpG islands) but depleted in north shelf regions (Additional file 4: Fig. S7B and C, FDR ≤ 0.05). In addition, we found that CpGs associated with shared-tissue *cis*-mQTL exhibited a greater than expected by chance enrichment of informative CpGs (687 out of 812 unique CpGs in shared-tissue *cis*-mQTLs, 85%, 10,000 permutations, *p* < 1 × 10^−4^), further substantiating that site-specific DNAm correlation between tissues are influenced, in part, by genetic variation (Additional file 5: Fig. S8).

### Tissue-specific differential DNAm was consistent across cohorts

**Fig. 4:**
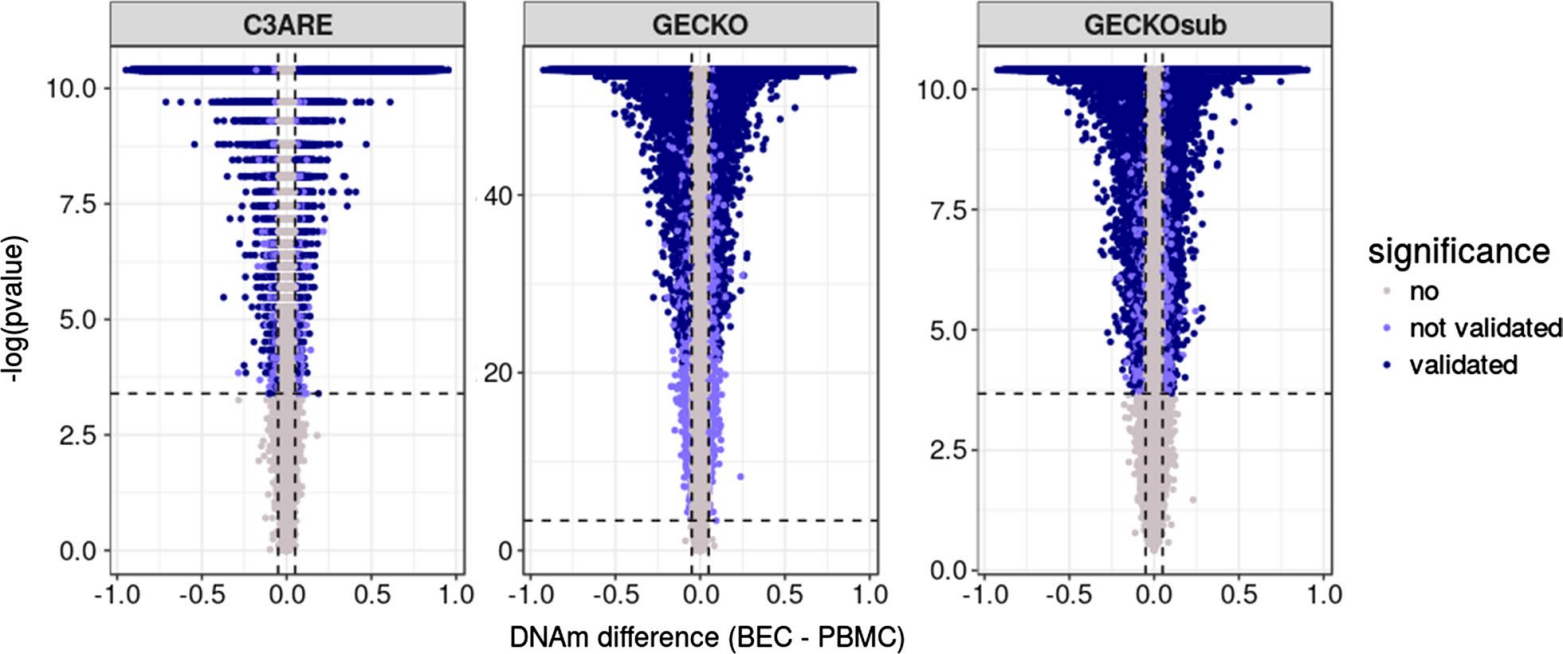
Tissue-specific differential DNA methylation was consistent across cohorts. Volcano plots of differential methylation analysis (run using a paired Wilcoxon signed-rank test) between BEC and PBMC tissues for C3ARE, GECKO and GECKOsub datasets. Vertical lines represent an effect size threshold of > 0.05 for absolute mean difference between tissues (BEC–PBMC) and the horizontal line represents the nominal *p* value corresponding to an FDR < 0.05 in each cohort. CpGs in dark purple met the effect size and significance cut-offs independently in all three datasets (139,662 CpGs). GECKO − log *p* values were ~ 5X greater than that of GECKOsub and C3ARE likely due to sample size differences between datasets (*n* = 79, *n* = 16, *n* = 16, respectively); y-axes were left unstandardized to display trends within each cohort

Taking further advantage of our matched tissue design, we subsequently assessed differential DNAm between PBMCs and BECs at individual CpGs for both cohorts. In the GECKO samples, 36% of CpGs (150,647) were differentially methylated between matched BECs and PBMCs (Wilcoxon signed-rank test; FDR ≤ 0.05 and delta beta ≥ 0.05). The number of significant differentially methylated sites was not greatly affected by sample size differences as GECKOsub had similar findings with 36% of sites exhibiting differential DNAm (149,094 CpGs, with 148,767 sites overlapping with GECKO). Similarly, in C3ARE, 38% of CpGs (157,992) were significantly differentially methylated (Wilcoxon signed-rank test; FDR ≤ 0.05 and delta beta ≥ 0.05). The overwhelming majority of these CpGs (139,662) were differentially methylated in the same direction in GECKO, GECKOsub and C3ARE (Fig. 4). Of these sites, 102,203 (73%) had greater average DNAm in PBMCs and 37,459 (27%) had greater average DNAm in BECs. This corresponded with a greater median DNAm across all PBMC probes (68%, 68%) as compared to all BEC probes (47%, 50%) in both C3ARE and GECKO, respectively.

### Differentially methylated sites were common in previously published EWAS findings

**Fig. 5:**
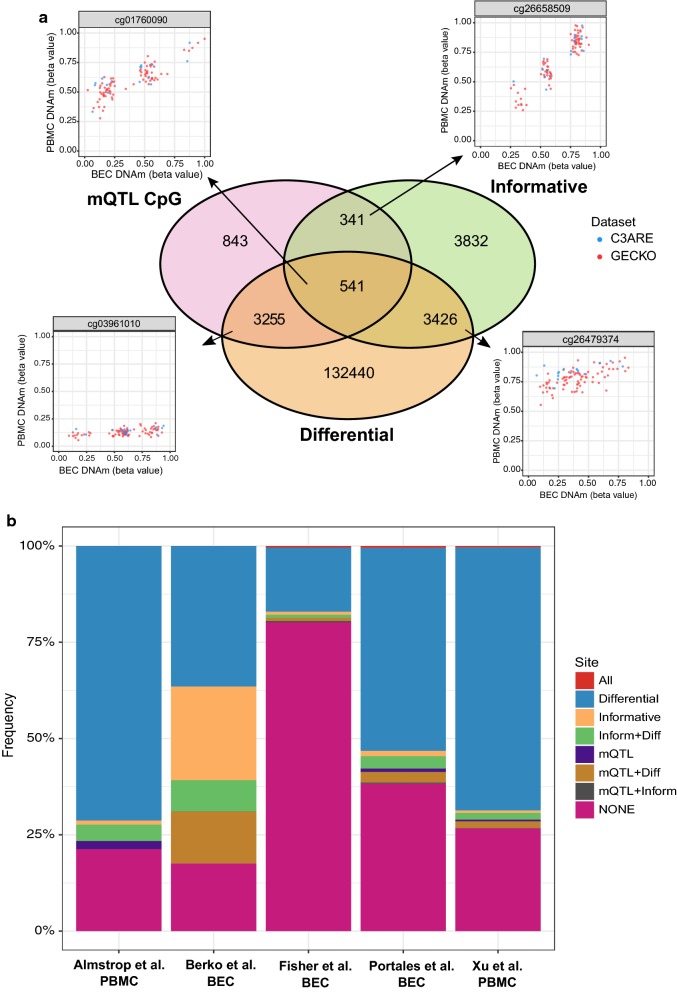
Overlap and representation of identified CpGs in previously published pediatric EWAS findings. **a** Venn diagram of CpGs identified as informative, differentially methylated between tissues, or underlying our set of validated *cis*-mQTL. Scatterplots display three representative CpGs from the pairwise intersections between categories. **b** Stacked bar plot showing proportion of CpGs of each defined category represented in significant CpGs of various pediatric EWAS publications in BECs or PBMCs (All = all categories; Differential = differentially methylated between tissues; Informative = informative CpG; Inform + Diff = informative and differential; mQTL = CpG associated with mQTL; mQTL + Diff = mQTL CpG and differential; mQTL + Inform = mQTL CpG and informative; None = not in any of the listed categories)

To provide a granular categorization of CpGs measured on the 450K array, we overlapped CpGs that were identified as a) informative (i.e., variable across individuals and correlated between BECs and PBMCs) (8140), b) differentially methylated between matched tissues (139,662), or c) under genetic influence (4980; i.e., number of unique CpGs associated with validated *cis*-mQTL) across both GECKO and C3ARE cohorts. Of all CpGs associated with *cis*-mQTL, 17.7% were informative and 76.2% were differentially methylated (Fig. 5a). However, in CpGs associated with cross-tissue *cis*-mQTL (812 unique CpGs in total), 84.6% were informative and 58.8% were differentially methylated.

We then applied this categorization scheme to previously reported EWAS findings performed in pediatric BEC or PBMC tissues to provide an example of how the classification of CpGs can aid in the interpretation of such studies. We selected five published studies that used the 450K array in pediatric BECs or peripheral blood to assess DNAm variation associated with puberty, aging in early life, childhood psychotic symptoms, fetal alcohol spectrum disorder and autism spectrum disorder [61–65]. By implementing our CpG classification scheme on their respective list of significant EWAS hits, we found that *cis*-mQTL, as identified here, accounted for 0.02–13.5% of significant CpGs reported in these five studies. Differentially methylated CpGs comprised the most represented type of CpG across all five studies with only one study demonstrating an overlap of 24.3% with our identified informative sites (Fig. 5b; Additional file 6: Table S2) [65]. This suggested that the majority of DNAm associations identified in these EWASs were likely specific to peripheral blood or BECs, rather than shared across tissues. Finally, we tabulated our CpGs classifications across all 419,507 DNAm probes assessed in our study in order to serve as a resource for researchers wishing to compare their own EWAS results (Additional file 7). Collectively, these findings reveal the importance of considering DNAm variability and concordance between tissues, as well as genetic influences on these patterns, when interrogating and interpreting EWAS findings from pediatric peripheral tissues.

## Discussion

In this study, we comprehensively compared genome-wide DNAm in BECs and PBMCs using matched samples from two independent pediatric cohorts. Moreover, we leveraged the strength of paired DNAm and genotyping profiles to define *cis*-mQTL across the genome and assess the influence of local genetic variation on DNAm variability and tissue concordance. Our findings showed that at the genomic and site-specific level, BECs had greater inter-individual DNAm variability over PBMCs, with highly variable CpGs more likely to be positively correlated between the matched tissues. In our subsequent *cis*-mQTL analyses, we observed distinct genetic influences on tissue-specific DNAm and confirmed that a sizeable proportion of shared DNAm patterns between tissues resulted from allelic variation. Finally, we provided a classification framework for the post hoc examination of EWAS associations and examined the representation of our categorized CpGs in published EWAS findings performed in pediatric BECs and PBMCs.

Our findings highlighted extensive differences in DNAm patterns between tissues and thus the importance of tissue selection when designing an EWAS. To a large extent, EWAS tissue selection in early life cohorts is guided by two factors. Firstly, ease of collection is particularly important in this age range and may restrict tissue availability. Buccal swabs are less invasive than intravenous puncture, and the latter contributes to participation refusal in pediatric cohorts [66]. Secondly, the relevance of the tissue to the phenotype or exposure being tested represents an important consideration for all EWAS analyses, irrespective of age. As peripheral blood represents a circulating tissue with broad immune and inflammatory functions, it might be more relevant to a wider range of health phenotypes than BECs. However, another hypothesis posits that tissues that arise from the same germ layer are more epigenetically similar and thus might be a preferred choice for surrogate tissue selection [67]. For example, in comparison with blood, it has been proposed that BEC DNAm may more closely reflect brain DNAm than blood DNAm, as both derive from the ectodermal germ layer [32, 65]. Adding to the complexity of this issue, we found that BEC DNAm had significantly greater inter-individual variability than PBMC DNAm at the genome-wide level and at the site-specific level, a finding consistent with adult BECs and PBMCs [20]. Having a higher proportion of variable CpGs might be desirable for EWAS analyses as testing any tissue with little inter-individual DNAm variation would naturally limit effect sizes. From this perspective, BECs might represent a more appropriate choice of peripheral tissue for population-based epigenetic studies over PBMCs. However, it is worth noting that while we did correct for cellular heterogeneity in both tissues using bioinformatic deconvolution approaches, the higher proportion of variable CpGs in BECs may, to some extent, be attributed to the increased diversity of cell types or residual cellular heterogeneity in BECs over PBMCs (i.e., epithelial and hematopoietic in the former and entirely hematopoietic in the latter) [68].

Taking advantage of our matched sample design, we were able to rigorously interrogate the extent of correlation between DNAm signatures of BECs and PBMCs. CpGs with greater variability were more likely to be correlated between matched tissues, as best exemplified by the 8140 informative sites we identified. These may aid in the inference of unmeasured PBMC or BEC DNAm (when the other tissue is measured) as well as for prioritization of sites for cross-tissue replication. In the latter case, cross-tissue replication typically involves the generation of candidate gene lists in accessible tissues for validation in less available tissues, such as postmortem samples, an approach which can boost confidence in identified associations [69–71]. There was a substantial overlap (45%) between our informative sites and those previously published in matched adult blood and brain tissues [31]. However, we found only 1.9% of total measured CpGs to be informative by our measures and thresholds as compared to 9.7% found in the previous analyses of adult samples from our laboratory [31]. These quantitative differences might be due to a number of reasons, with the most likely being that the blood–brain informative sites were identified using a single cohort, while our blood–buccal informative sites were filtered down to sites that were common across both GECKO and C3ARE cohorts; other explanations may be methodological (i.e., slight differences in analytical thresholds derived from empirical testing) or biological (i.e., blood may be more epigenetically similar to brain tissue than to BECs, resulting in more informative sites). An in-depth analysis of such cross-tissue comparisons between pediatric and adult samples, ideally by means of longitudinal sampling of DNAm, may help elucidate such sources of tissue variation across the lifespan.

Integration of genetic and epigenetic information may further clarify the relative contribution of genetic and environmental factors on inter-individual DNAm variability. We found that genetic variation contributed to both inter-individual DNAm variation within a tissue, as well as common DNAm variation between tissues. This is in general agreement with previous findings that show that many—but not all—mQTLs have consistent effects across tissues and human populations and are generally depleted in genomic regions which tend to have low DNAm variability such as promoters and CpG island but enriched in more variable intergenic and intragenic regions [41, 43, 44, 46, 72]. It is currently unclear why we observed more BEC-specific mQTL in our matched design as compared to PBMC-specific or cross-tissue mQTL. The most likely explanation is that BECs contained more validated *cis*-mQTL due to greater inter-individual DNAm variability. It is also tempting to speculate that allelic variation contributes more strongly to DNAm in BECs over PBMCs, because blood DNAm might be more plastic and responsive due to the role of blood cells in the immune system [73–75]. For example, changes in genome-wide transcriptional programs and DNAm profiles are observed in response to an inflammatory stimulus in blood leukocytes, which could be incongruent with a high degree of fixed, genetically driven DNAm patterns in these cells [73–75]. In a more complicated paradigm, DNAm variation may be best explained by the interaction of both genetic and environmental factors ((G × E) interactions), as previously demonstrated in blood-based DNAm profiles [45, 47].

As touched upon in several recent reviews, genetic contribution to DNAm might be more prominent in shaping the DNA methylome than initially anticipated, and thus affect the analysis and interpretation of EWAS findings [1, 76]. To illustrate this, we tested for the presence of our categorized CpGs in published EWAS findings. Notably, we found that while most identified EWAS associations may be distinct to the tissue in which they were examined, in some instances, these associations may be reflected across multiple tissues and/or under genetic influence. For example, we observed CpGs associated with autism spectrum disorder to contain the highest proportion of *cis*-mQTL. While there might be a number of reasons for this, it is possible that the proportion of genetically influenced CpGs found in an EWAS may be proportional to the heritability of the phenotype under examination, although such hypotheses will require rigorous testing in large cohorts across a diverse spectrum of phenotypes with and without heritable contributions. Furthermore, it is difficult to discern whether having a high proportion of mQTL in EWAS analyses is favorable or not. Previous work has shown that the majority of variably methylated regions are best described by an interaction of both genetic and environmental factors [47, 71]. Emerging findings from neonatal blood samples have additionally shown that the bulk of variable DNAm sites are best accounted for by either additive (G + E) or interaction (G × E) models, suggesting that environmental influences on DNAm may be further delineated with the inclusion of genotype information [47, 77]. As such, any mQTL CpGs found in an EWAS may offer alternate interpretations to phenotypic associations with DNAm and would require further investigation for potential gene-environment effects.

It is worth noting that our study had a few inherent limitations. Firstly, in both GECKO and C3ARE cohorts, PBMCs were collected from individuals at a slightly later time point than BECs, resulting in an age-related difference (0–1.5 years for C3ARE; 0.5–2.3 years for GECKO) between matched tissues, which may have affected analyses of DNAm variability. However, we anticipate that age-related differences in DNAm variability are relatively small compared to tissue-specific differences as our findings are consistent with previous work performed on age-matched tissues in adults [20]. Another limitation was the relatively small sample size of our cohorts, which may have inflated type II error rates. We also chose to not assess distal genetic effects on DNAm (i.e., *trans*-mQTL) due to the increased multiple testing burden, but rather prioritized *cis*-mQTL as previous work has suggested these may be more functionally linked to nearby CpGs [47, 59, 60]. As well, previous work in blood has shown that the proportion of DNAm variance explained by *trans*-mQTL is much lower than that of *cis*-mQTL [46]. For these reasons, we examined SNPs that were directly measured and not imputed, as performed in other pediatric mQTL analyses, within a 5 kb window [47, 78]. As a result, we likely underestimated the number of mQTL present in our tissues. Future work using large cohorts will be required to clarify the contribution of distal genetic variants to DNAm in other peripheral tissues. In addition, our mQTL findings were limited by the coverage of the 450K array, which interrogates less than 2% of all DNAm sites across the genome, although this includes 94% of all mapped CpGs islands. As such, it is generally biased toward CpG-dense promoter regions, which typically have limited inter-individual and inter-tissue variation [21, 79–81]. Finally, while we found the Houseman blood deconvolution method to perform well in our cohorts, evidence of substantial DNAm changes across the lifespan, especially during early childhood, necessitates the refinement of cell deconvolution methods, including adjusting for age, to allow for more nuanced estimation of cell types in early life [21, 22, 58].

## Conclusions

The work here presents a comprehensive assessment of local genetic influences on DNAm in matched BECs and PBMCs, as well as a characterization of DNAm variability and concordance between paired pediatric tissues. Moreover, our results highlight a number of possible considerations for EWAS analyses, including the potential enrichment of mQTL findings following prefiltering to variable CpGs to reduce multiple test barriers and possible strategies to facilitate in-depth curation of EWAS hits. Such post hoc examination of significant differentially methylated CpGs will hopefully support the interpretation of EWAS findings and aid in the prioritization of candidate associations for functional validation.

## Methods

### Study cohorts and tissue samples

Matched tissues were obtained from a subset of two separate pediatric cohorts. Specifically, a subset of samples from the previously described C3ARE (Cleaning, Carrying, Changing, Attending, Reading and Expressing) cohort were collected from 16 individuals (8 females; 50%) aged 3–5 years (age range 3.6–4.2 years (BEC) and 4.5–5.2 years (PBMC)) from Vancouver, British Columbia [82]. The GECKO cohort samples (Gene Expression Collaborative Kids Only) comprised of 79 individuals (36 females; 46%) aged 6–13 years (age range 6–11 years (BEC) and 7–13 years (PBMC)) also from Vancouver, British Columbia. Birth dates were not available for all GECKO participants; age in years was recorded at the BEC sample collections. In both cohorts, the majority of BEC samples were collected at the first visit and PBMCs were collected at a later date. In the C3ARE cohort, follow-up visits ranged from 7 days to 1.5 years, with three pairs of matched BECs and PBMCs being collected on the same day. In the GECKO cohort, the follow-up visits at which peripheral blood was collected ranged from 6 months to 2.3 years after the initial visit. Demographic descriptors of both cohorts are provided in Table 1. All experimental procedures were conducted in accordance with institutional review board policies at the University of British Columbia. Written informed consent was obtained from a parent or legal guardian and assent was obtained from each child before study participation. For both cohorts, BECs were collected using the Isohelix Buccal Swabs (Cell Projects Ltd., Kent, UK) and stabilized with Isohelix Dri-Capsules for storage at room temperature prior to DNA extraction, as previously described [64]. Whole blood was collected into Vacutainer^®^ CPT™ Cell Preparation Tubes (Becton, Dickinson and Company, NJ, USA) and PBMCs were isolated following centrifugation, washing and resuspension into R10 media (Sigma-Aldrich, MO, USA), as previously described [83]. PBMC pellets were frozen and stored at − 80 °C until DNA extraction.

### DNA isolation and DNA methylation arrays

Genomic DNA from stabilized buccal samples was isolated using Isohelix Buccal DNA Isolation Kits (Cell Projects Ltd., Kent, UK) and was purified and concentrated using DNA Clean & Concentrator (Zymo Research, CA, USA). Genomic DNA was extracted from PBMC pellets using the DNeasy kit (Qiagen, MD, USA). DNA yield and purity were assessed using a Nanodrop ND-1000 (Thermo Fisher Scientific, MA, USA). Bisulfite conversion of DNA (750 ng) was performed using the Zymo Research EZ DNA Methylation Kit (Zymo Research, CA, USA). Samples were subsequently randomized, and 160 ng of bisulfite-converted DNA was applied to the Illumina Infinium HumanMethylation450K Beadchip (450K) array, as per manufacturer’s protocols (Illumina, CA, USA) [79].

### DNA methylation array data quality control and normalization

Data from each cohort were analyzed separately. Specifically, raw intensity values from the DNAm arrays were imported into Illumina GenomeStudio V2011.1 software and subjected to initial quality control checks for array staining, extension and bisulfite conversion followed by color correction and background adjustment using control probes contained on the 450K array. Data were exported from GenomeStudio as beta values which represent the estimated DNAm level based on a ratio of intensities between methylated and unmethylated alleles, with beta values ranging from 0 (unmethylated) to 1 (fully methylated). Subsequent processing and analysis were performed in R Version 3.2.1 (http://www.r-project.org). Profiles from 65 probes targeting single nucleotide polymorphisms (SNPs) were used to ensure matched tissue samples originated from the same individual. The 65 SNP probes were subsequently filtered out of the dataset. Since the cohorts were not equally matched for sex, we removed sex chromosome probes (11,648) from both datasets. Additional probe filtering was performed in which poor performing probes including those with detection *p* values greater than 0.01 or probes with missing beta values in more than 2% of samples were removed (14,400 C3ARE, 13,374 GECKO). Reannotation of the Illumina 450K array was used to filter probes that are known to be polymorphic at the target CpG. Probes, which have non-specific in silico binding to the sex chromosomes, were also removed [84]. Final probe count after quality control probe filtering was 429,494 probes for C3ARE and 430,581 probes for GECKO. Following quality control processing, quantro determined quantile normalization to be inappropriate as the global DNAm distributions between the two distinct tissues were highly differential [85]. Beta Mixture Quantile dilation (BMIQ) normalization was performed to remove differences between Type I and Type II probes on the 450K array, yielding normalized DNAm [48].

### Cell type correction of DNA methylation data

The effects of cellular heterogeneity on DNAm measures were removed from PBMC and BEC samples in both cohorts. Specifically, blood cell type proportions were estimated for the PBMC samples using the established Houseman blood deconvolution method [49, 50]. This blood deconvolution algorithm has been previously used in pediatric blood DNAm profiles where it was shown to perform reasonably well [23]. To test whether this was indeed also true in our GECKO and C3ARE samples, we assessed the appropriateness of the Houseman probeset panel in our pediatric blood samples compared to adult blood profiles [49, 86]. We downloaded the original adult blood DNAm dataset (Reinius) on which the Houseman method was trained (Accession# GSE35069) and filtered to 500 probes used in the algorithm that were common across all GECKO, C3ARE (following preprocessing) and Reinius samples [86]. Given that this Houseman signature comprises 600 statistically related probes, 500 of which passed quality control in both GECKO and C3ARE, we chose to use two commonly used analytical approaches, principal component analysis (PCA) and hierarchical clustering, to determine the relationship of methylation states between cohorts in the data. PCA showed an overlap of child and adult PBMC profiles in the two top-ranking PCs (accounting for 98% of the DNAm variance of the Houseman probeset panel) and similarly, adult samples did not cluster separately from child samples in the hierarchical clustering analysis. Collectively, these findings suggested that DNAm at CpGs used in the Houseman deconvolution signature were similar between adult and child blood samples (Additional file 8: Fig. S1). Given that no cell deconvolution algorithm for buccal tissues exists and that buccal swabs, like saliva, are predominantly composed of BECs and leukocytes, we used a saliva-based deconvolution method which was designed to predict these cell types from underlying DNAm patterns [34, 68, 87]. Predicted cell proportions from both PBMC and BEC tissues were used to normalize cellular heterogeneity within each tissue using a regression-based strategy [51] (Additional file 9: Fig. S2). PCA was subsequently used to confirm that the correlation of estimated cell type proportions to DNAm variance within a tissue was minimal in the corrected 450K datasets (data not shown).

### Assessment of cross-tissue correlation, tissue-specific variability and tissue-specific differences in DNA methylation data

Prior to subsequent DNAm analyses, the corrected 450K datasets were filtered down to overlapping probes (419,507) between the GECKO and C3ARE cohorts. Probewise cross-tissue Spearman’s correlations were calculated on beta values between the matched PBMC and BEC tissues. Inter-individual variability of each CpG was calculated as the range between the 10th and 90th percentile beta values for each CpG, referred to as “reference range” [88]. This method captures variability across the bulk of samples while being largely robust to outlier samples.

In order to assess sample size-related differences in our DNAm analyses between GECKO and C3ARE, we performed 100 trials of Monte Carlo simulations. Specifically, we randomly subsampled the GECKO cohort to the equivalent size as the C3ARE cohort (*n* = 16 individuals) 100 times and reran the cross-tissue correlations and reference range calculations on the subsamples. We reported the average correlation coefficients, *p* values and references ranges from the 100 trials, which we refer to as “GECKOsub.”

Paired Wilcoxon signed-rank tests were used to compare global differences in reference range between matched BEC and PBMC samples. Fligner–Killeen tests were used to compare probewise variability differences in each of the cohorts. Using previously published methods, we aimed to identify informative sites between BECs and PBMCs, which we defined as CpGs that are both variable across individuals and highly correlated between both tissues [31]. To identify informative sites, we first subset each cohort down to CpGs with a reference range greater than 0.10 in both tissues. We subsequently ran a beta mixture model on Spearman correlation rho values generating two Gaussian distributions, which separated out a group of highly concordant CpGs (Additional file 10: Fig. S3). The Spearman rho distributions in this set of highly correlated CpGs was used to define a threshold correlation coefficient, the cutoff being two standard deviations lower than the mean of the distribution. In the GECKO cohort rho > 0.47 was determined as the threshold and in the C3ARE cohort, rho > 0.32 was determined as the threshold. We also set a minimum reference range of 0.05 in both tissues to exclude CpGs with little inter-individual variation.

Finally, we identified CpGs which were differentially methylated between tissues by running Wilcoxon signed-rank tests across all probes in the C3ARE, GECKO and GECKOsub datasets. For all tests, the resulting *p* values were adjusted using the Benjamini–Hochberg (BH) false discovery rate (FDR) method [89]. CpGs which passed an FDR < 0.05 and an effect size threshold, delta beta > 0.05, independently in all three datasets, C3ARE, GECKO and GECKOsub, were classified as “differential sites.”

### SNP genotyping arrays

In the GECKO cohort, DNA for genotyping was collected from saliva samples of 63 individuals using the Oragene OG-500 DNA all-in-one system as per manufacturer’s protocol (DNA Genotek Inc, ON, Canada). In the C3ARE cohort, genomic DNA for genotyping was obtained from PBMC samples as described above. Genotyping data was measured at 588,454 SNP sites using the Illumina Infinium PsychChip BeadChip (PsychChip), as per manufacturer’s protocols (Illumina, CA, USA). Content for the PsychChip includes 264,909 proven tag SNPs found on the Infinium Core-24 BeadChip, 244,593 markers from the Infinium HumanCoreExome BeadChip, and 50,000 additional markers associated with common psychiatric disorders.

### Preprocessing of SNP genotyping data and PCA analyses for genetic ancestry

Quality control prepreprocessing of Illumina Infinium PsychChip data was performed separately for each cohort according to recommended guidelines [90]. Specifically, SNPs with a low 10th percentile GenCall score or with a low average GenCall score were filtered out. Additionally, SNP probes located on mitochondrial DNA, on sex chromosomes or without chromosome labels were removed. After probe filtering, final SNP probe counts for the C3ARE and GECKO datasets were 550,200 and 547,662, respectively. To test for difference in genetic ancestry between the two cohorts, we ran all samples in PCA, using the 542,699 SNPs called for every individual in both processed datasets. Genetic ancestry was not found to differ significantly between the cohorts (Additional file 11: Fig. S4), as determined by Wilcoxon ranked-sum test of GECKO vs C3ARE in PC1 scores (*p* = 0.8) and PC2 scores (*p* = 0.4). Therefore, genetic ancestry was not considered in further analyses.

### Cis-mQTL analyses

We ran *cis*-mQTL analyses in each cohort separately, using GECKO as the discovery cohort and C3ARE as the validation cohort. In the GECKO cohort, PsychChip data were filtered after quality control to remove any SNP probes containing missing values in 5% of all samples, leaving 560,770 SNPs. In addition, SNPs with a minor allele frequency less than 5% or not in Hardy–Weinberg equilibrium were removed. Remaining SNPs (249,835) were then numerically coded, as 1, 2, or 3, for correlational analyses. Therefore, all SNPs used in mQTL analyses were directly measured on array, rather than generated through imputation. CpGs with a reference range of less than 5% were removed from mQTL analysis; this was performed separately in each tissue, leaving 131,706 CpGs in PBMCs and 210,784 CpGs in BECs. Finally, SNP–CpG pairs less than 5 kb apart were tested as mQTL using Spearman correlations. We selected a 5 kb window as previous mQTL analyses using whole genome bisulfite sequencing data reported that associations between SNP–CpG pairs are more likely to be causal within a 5 kb window [47, 59, 60, 91, 92]. In GECKO, a total of 165,591 unique SNP-CpG pairs in PBMC and 261,739 unique SNP-CpG pairs in BEC were interrogated for associations between DNAm and allelic variation; this included 145,222 SNP-CpG pairs tested in both tissues. Pairs with FDR ≤ 0.05 and DNAm change per allele ≥ 2.5% were designated as *cis*-mQTL hits and followed up for validation in the C3ARE cohort [93]. For validation testing in the C3ARE samples, SNP-CpG pairs were further filtered to exclude those with SNPs that were (a) not present in the filtered C3ARE PsychChip data or (b) monomorphic or had less than 2 heterozygotes in the C3ARE samples. The mQTL analyses were repeated in the C3ARE data. SNP-CpG pairs with FDR ≤ 0.05 and DNAm change per allele ≥ 2.5% were designated as validated *cis*-mQTL hits and followed up in subsequent analyses. All genotyping and DNAm data were analyzed using the human assembly GRCH37 (hg19) genome build. All SNPs are reported on the (+) strand, according to standard practices in the field.

### Representation of identified sites in published EWAS findings

In order to relate our results to published EWAS findings performed in pediatric cohorts, we selected five published studies which used the 450K array to measure DNAm profiles in pediatric BECs or peripheral blood. Specifically, these studies examined DNAm variation associated with puberty, aging in early life, childhood psychotic symptoms, fetal alcohol spectrum disorder and autism spectrum disorder [61–65]. For each study, we downloaded the list of probes reported as significant and matched these probes to sites, which we identified as: (1) informative sites, (2) differential sites and/or (3) *cis*-mQTL-associated CpGs. For one study, in which differentially methylated regions (DMRs) were reported, we downloaded the dataset (Accession # GSE50759) and extracted individual probes underlying the DMRs [65].

## Additional files

**Additional file 1: Fig. S5.** Density distribution of Spearman’s correlation coefficient (Rho) across 419,507 CpGs in matched BEC and PBMC tissues for GECKO, GECKOsub, GECKOsub Averaged (mean of 100 trials of GECKOsub) and C3ARE datasets.

**Additional file 2: Table S1.** The number of CpG sites at various thresholds of Spearman’s correlation rho and reference range for C3ARE, GECKO and GECKOsub datasets.

**Additional file 3: Fig. S6.** Overlap of *cis*-mQTL identified in matched tissues of both C3ARE and GECKO cohorts, respectively.

**Additional file 4: Fig. S7.** Representation of A) 4980 CpGs underlying validated *cis*-mQTL, B) tissue-specific mQTL-associated CpGs and C) shared-tissue mQTL-associated CpGs across various genomic features. Bars show the fold-change between CpG count in each genomic region and the mean count of randomly selected CpGs in that same genomic feature, from 10,000 iterations. Error bars show standard error (* denotes significant enrichment or depletion at FDR ≤ 0.05) (S = South; N = North).

**Additional file 5: Fig. S8.** Stacked bar plot representing overlap of identified informative sites in BEC-specific, PBMC-specific and shared-tissue validated *cis*-mQTL.

**Additional file 6: Table S2.** The number of CpG sites of each defined category represented in reported significant hits of various pediatric EWAS publications.

**Additional file 7.** CpGs classifications across all 419,507 DNAm probes assessed in this study.

**Additional file 8: Fig. S1.** A) We used a data reduction method, PCA, to compare the 500 probeset Houseman deconvolution signature in adult PBMC samples (Reinius) and child PBMC samples of GECKO and C3ARE. We observed an overlap of adult and child PBMC profiles in PC1 and PC2 (cumulatively accounting for 98% of DNAm variance in Houseman signature). B) Hierarchical clustering of adult PBMC samples (Reinius) and our pediatric PBMC profiles across all 500 Houseman deconvolution probes showed no discernible clustering between adult and child samples. These findings suggest that the Houseman deconvolution signature of both adult and child PBMC samples are consistent.

**Additional file 9: Fig. S2.** Predicted proportions of cell types in A) PBMCs and B) BECs for both datasets before and after cell type correction (Mono = monocytes; Gran = Granulocytes).

**Additional file 10: Fig. S3.** Beta mixture modeling on Spearman correlation rho values between matched BECs and PBMCs for A) GECKO and b) C3ARE cohorts. The bimodal distribution of Spearman rho values indicated two underlying populations of CpGs, a set of uncorrelated CpGs (shown in red) and a set of right-skewed highly positively correlated CpGs (shown in green). Correlation coefficient threshold for informative CpGs were determined at two standard deviations minus the mean of the green Gaussian distribution (GECKO rho = 0.47; C3ARE rho = 0.32).

**Additional file 11: Fig. S4.** Principal component analysis of PsychChip genotyping profiles (542,699 SNPs) for C3ARE (shown in blue) and GECKO (shown in red) revealed that genetic ancestry did not differ significantly between the cohorts as determined by Wilcoxon ranked-sum test of GECKO versus C3ARE in PC1 scores (p = 0.8) and PC2 scores (p = 0.4).

## Abbreviations

DNAm: DNA methylation
BEC: buccal epithelial cell
PBMC: peripheral blood mononuclear cell
EWAS: epigenome-wide association study
CpG: cytosine–phosphate–guanine
G × E: gene-by-environment
mQTL: methylation quantitative trait loci
450K array: Infinium HumanMethylation450 array
SNP: single-nucleotide polymorphism
BMIQ: Beta MIxture Quantile dilation
PCA: principal component analysis
BH: Benjamini–Hochberg
FDR: false discovery rate
