# Supplementary figures and images for: Integration of DNA methylation patterns and genetic variation in human pediatric tissues help inform EWAS design and interpretation
Source: Epigenetics Chromatin. 2019 Jan 2;12:1. doi: 10.1186/s13072-018-0245-6 (PMC6314079; doi:10.1186/s13072-018-0245-6)

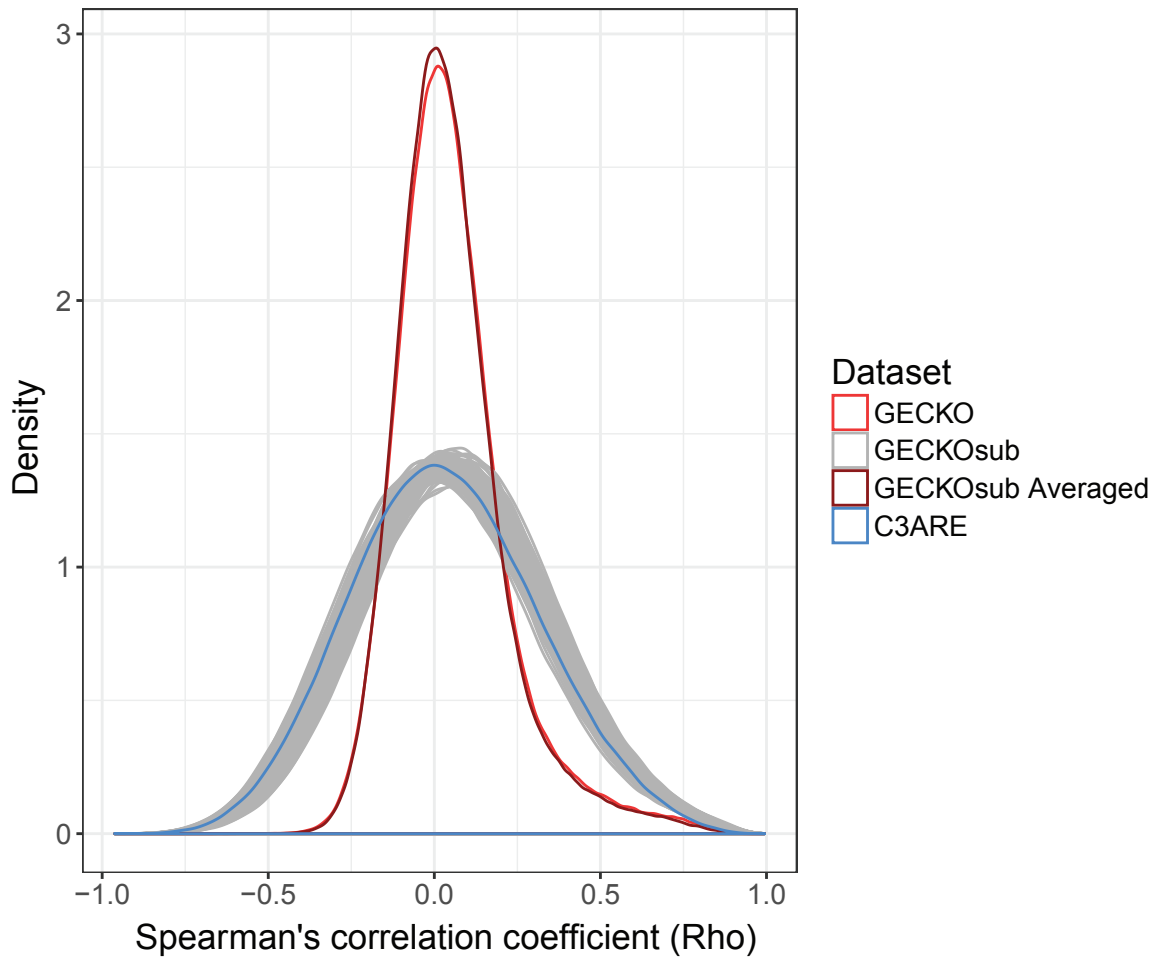

Supplement: Supplementary file 1 — Additional file 1: Fig. S5. Density distribution of Spearman’s correlation coefficient (Rho) across 419,507 CpGs in matched BEC and PBMC tissues for GECKO, GECKOsub, GECKOsub Averaged (mean of 100 trials of GECKOsub) and C3ARE datasets. [file 13072_2018_245_MOESM1_ESM.pdf]

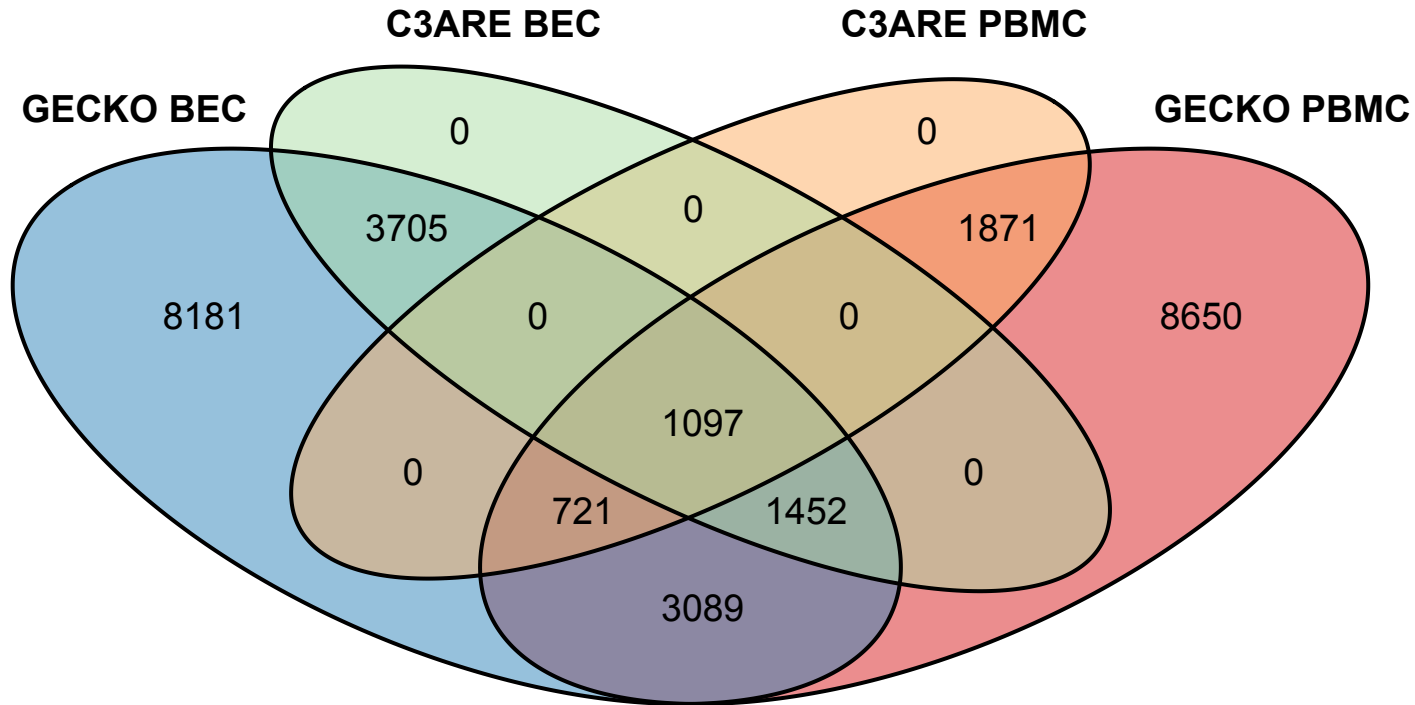

Supplement: Supplementary file 3 — Additional file 3: Fig. S6. Overlap of cis-mQTL identified in matched tissues of both C3ARE and GECKO cohorts, respectively. [file 13072_2018_245_MOESM3_ESM.pdf]

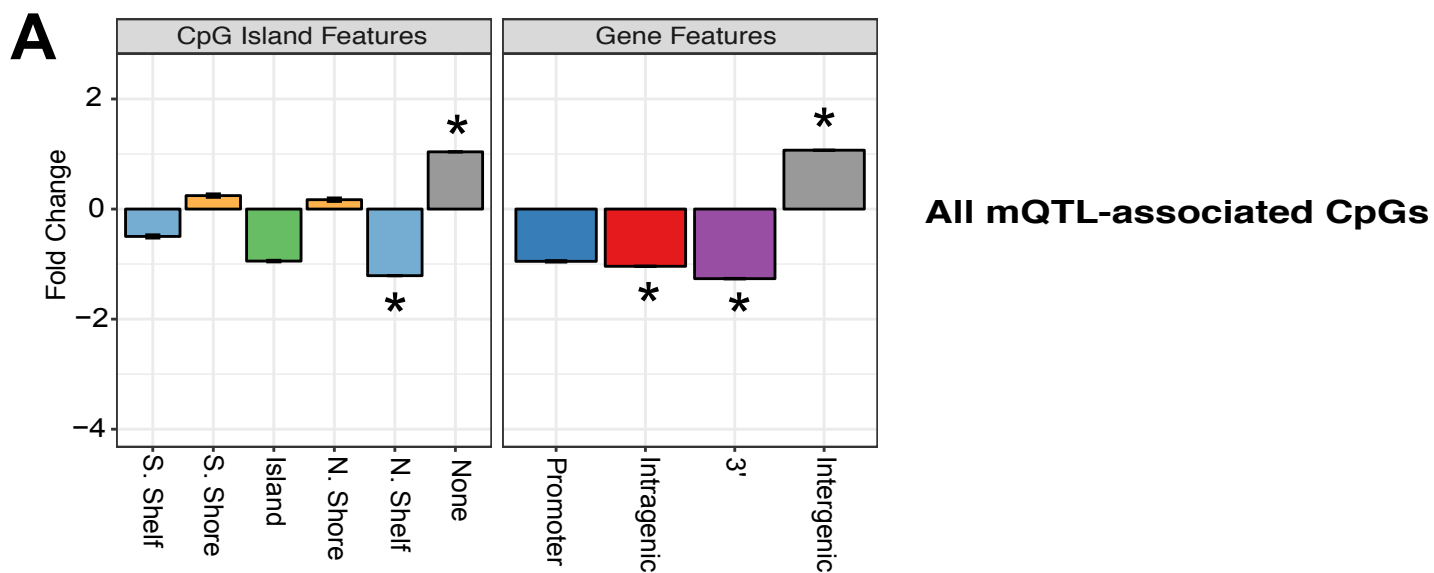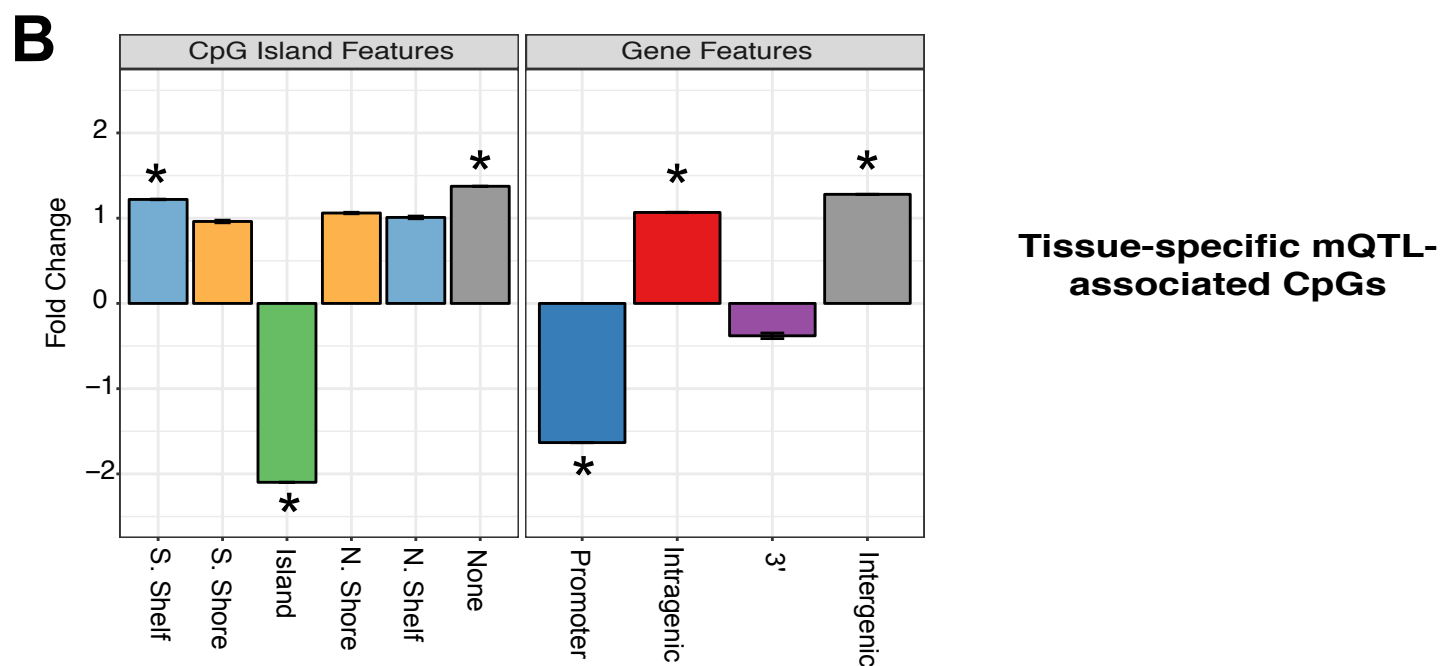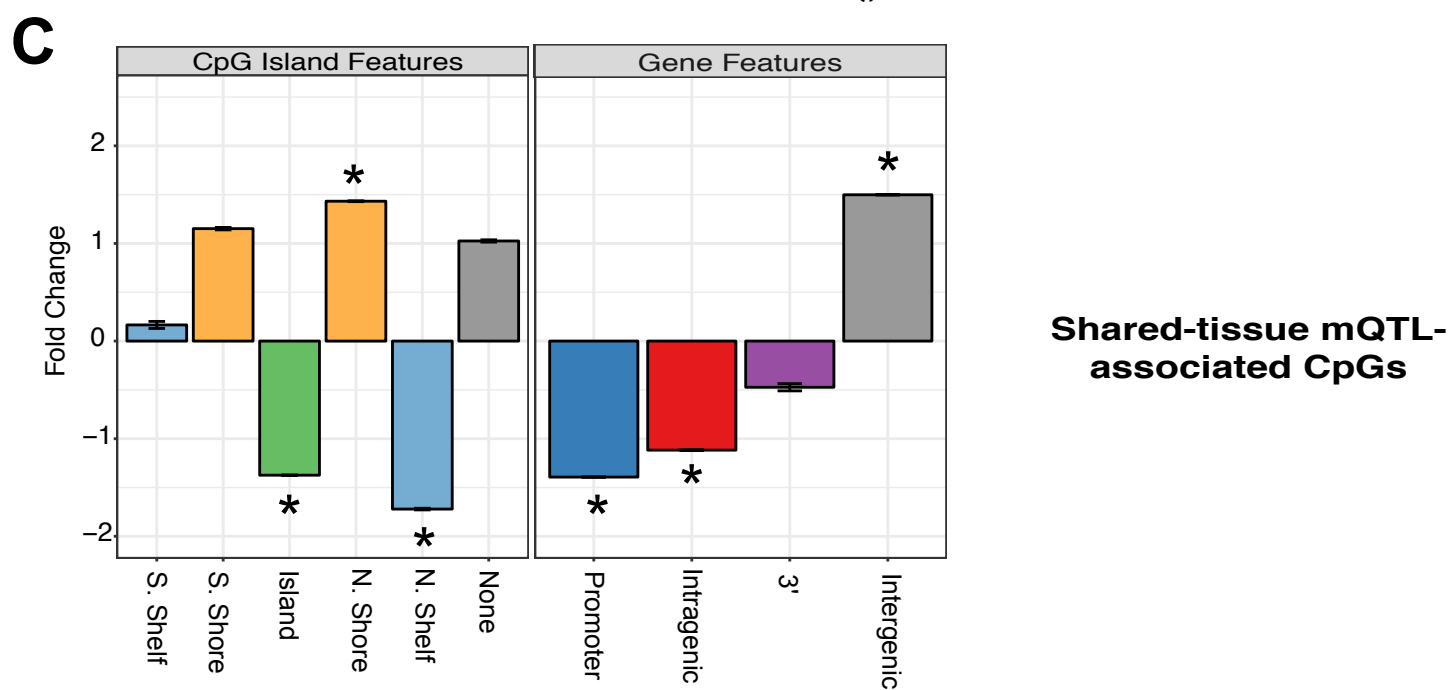

Supplement: Supplementary file 4 — Additional file 4: Fig. S7. Representation of A) 4980 CpGs underlying validated cis-mQTL, B) tissue-specific mQTL-associated CpGs and C) shared-tissue mQTL-associated CpGs across various genomic features. Bars show the fold-change between CpG count in each genomic region and the mean count of randomly selected CpGs in that same genomic feature, from 10,000 iterations. Error bars show standard error (* denotes significant enrichment or depletion at FDR ≤ 0.05) (S = South; N = North). [file 13072_2018_245_MOESM4_ESM.pdf]

mQTL count validated in C3ARE

Informative Site

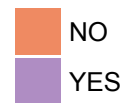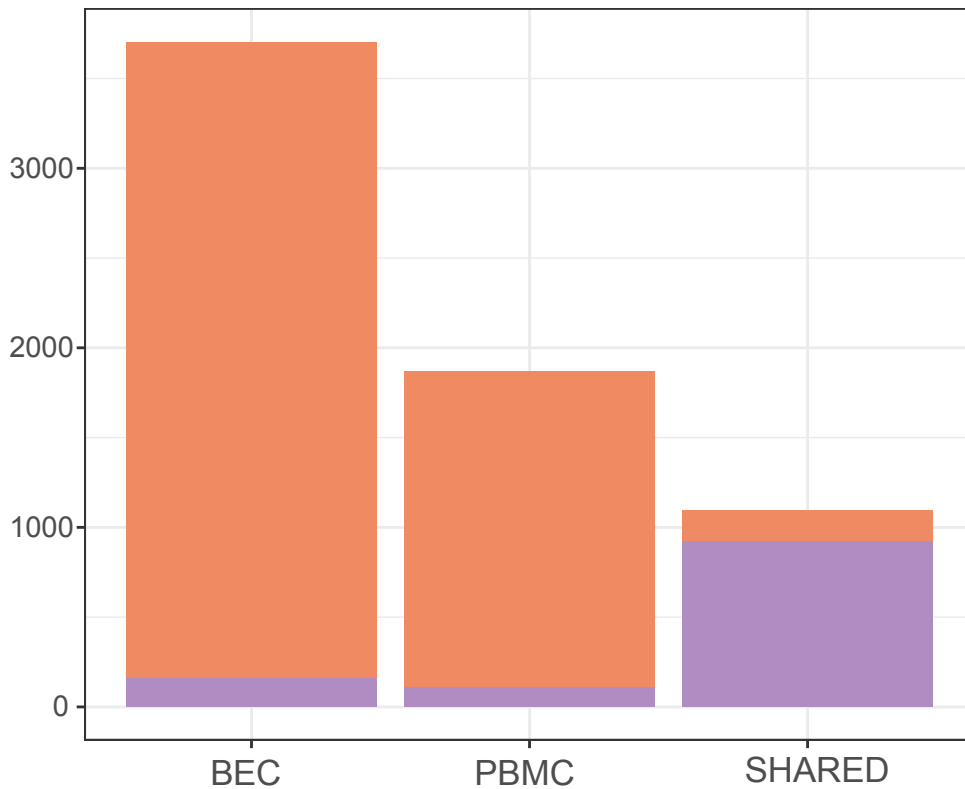

Supplement: Supplementary file 5 — Additional file 5: Fig. S8. Stacked bar plot representing overlap of identified informative sites in BEC-specific, PBMC-specific and shared-tissue validated cis-mQTL. [file 13072_2018_245_MOESM5_ESM.pdf]

**A**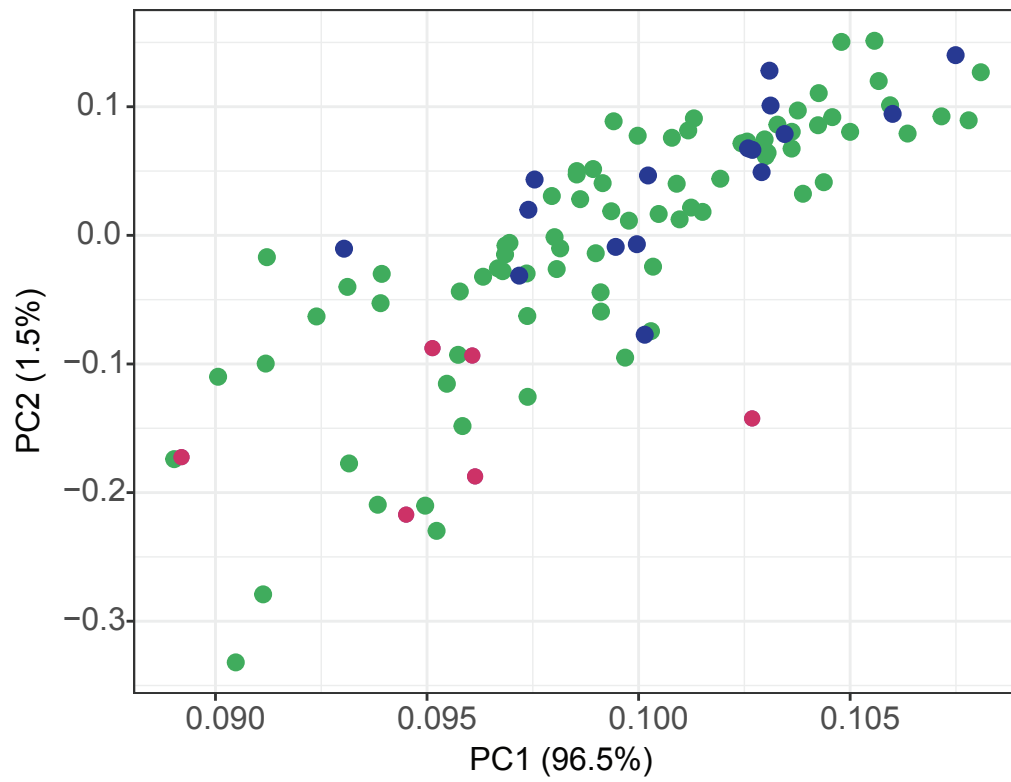**B**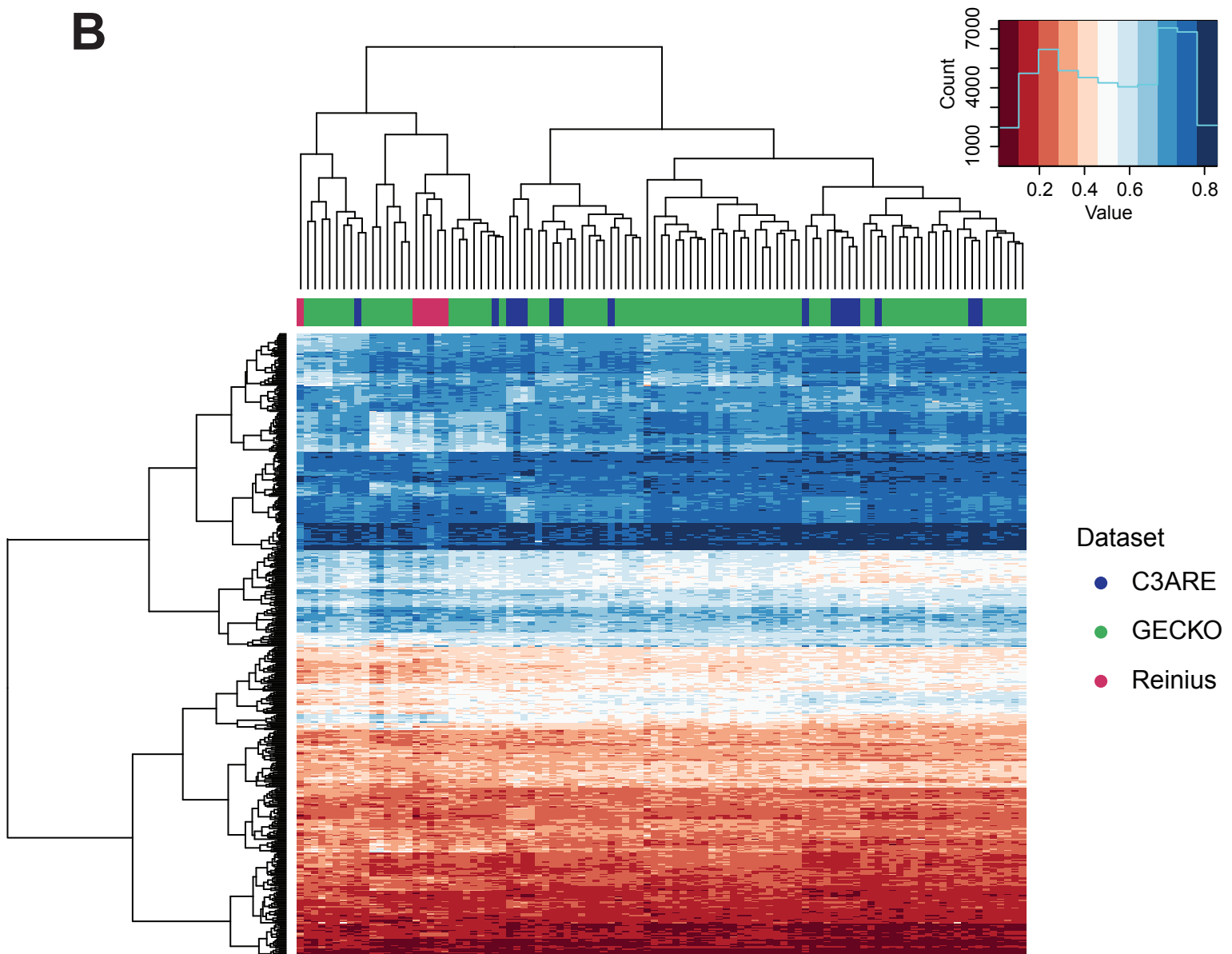

Supplement: Supplementary file 8 — Additional file 8: Fig. S1. A) We used a data reduction method, PCA, to compare the 500 probeset Houseman deconvolution signature in adult PBMC samples (Reinius) and child PBMC samples of GECKO and C3ARE. We observed an overlap of adult and child PBMC profiles in PC1 and PC2 (cumulatively accounting for 98% of DNAm variance in Houseman signature). B) Hierarchical clustering of adult PBMC samples (Reinius) and our pediatric PBMC profiles across all 500 Houseman deconvolution probes showed no discernible clustering between adult and child samples. These findings suggest that the Houseman deconvolution signature of both adult and child PBMC samples are consistent. [file 13072_2018_245_MOESM8_ESM.pdf]

**A**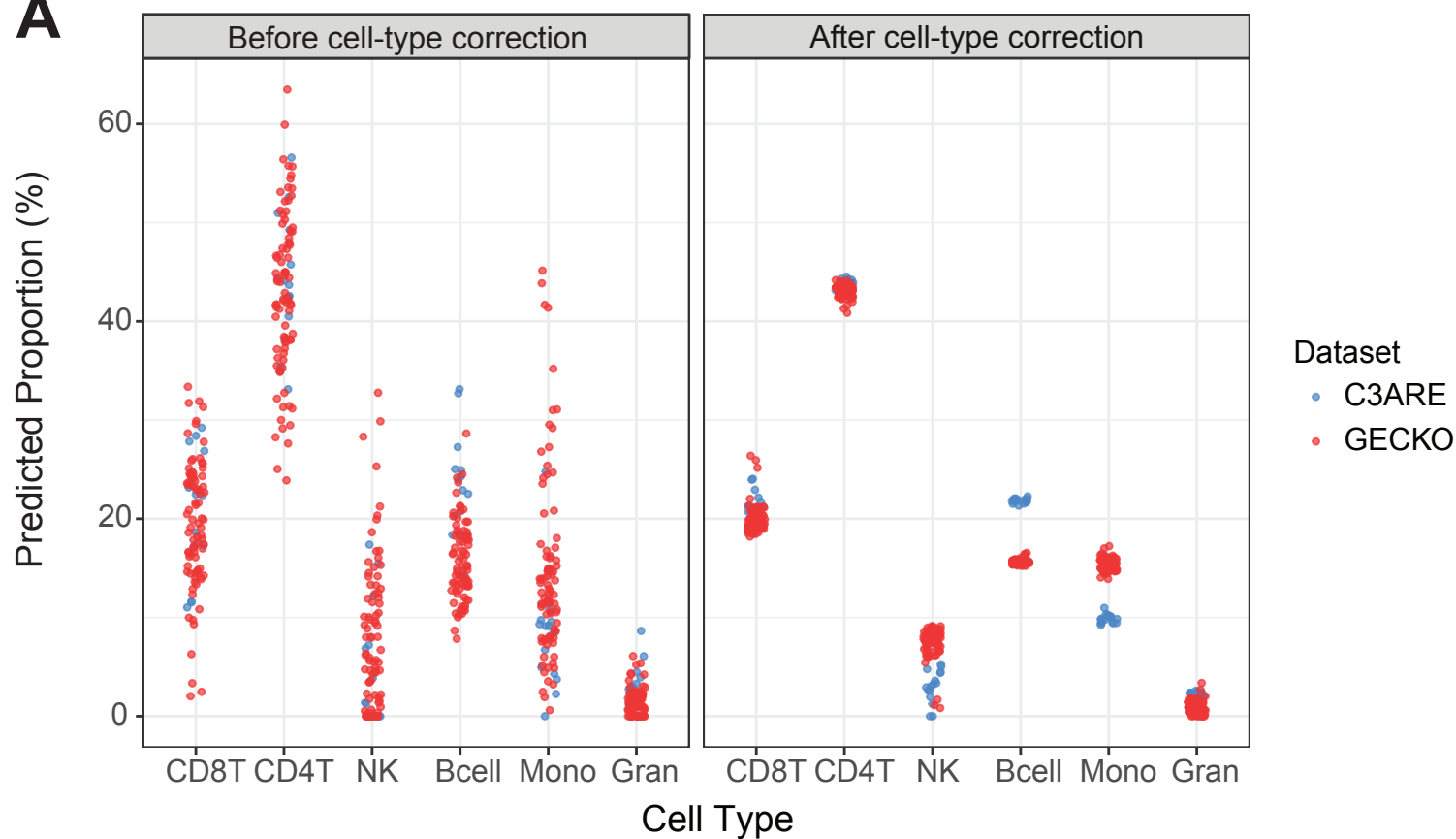**B**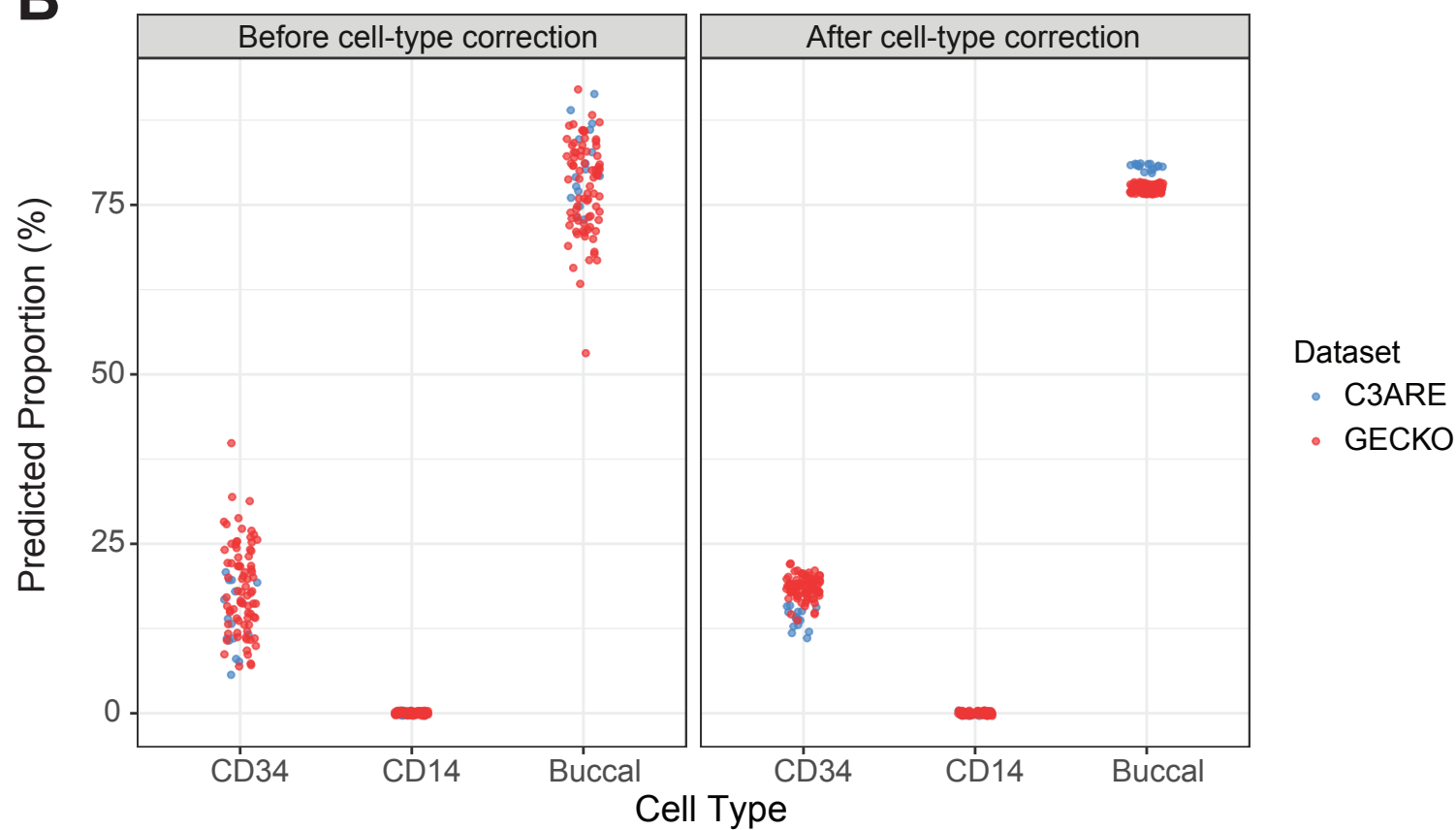

Supplement: Supplementary file 9 — Additional file 9: Fig. S2. Predicted proportions of cell types in A) PBMCs and B) BECs for both datasets before and after cell type correction (Mono = monocytes; Gran = Granulocytes). [file 13072_2018_245_MOESM9_ESM.pdf]

**A**

### GECKO Density Curves

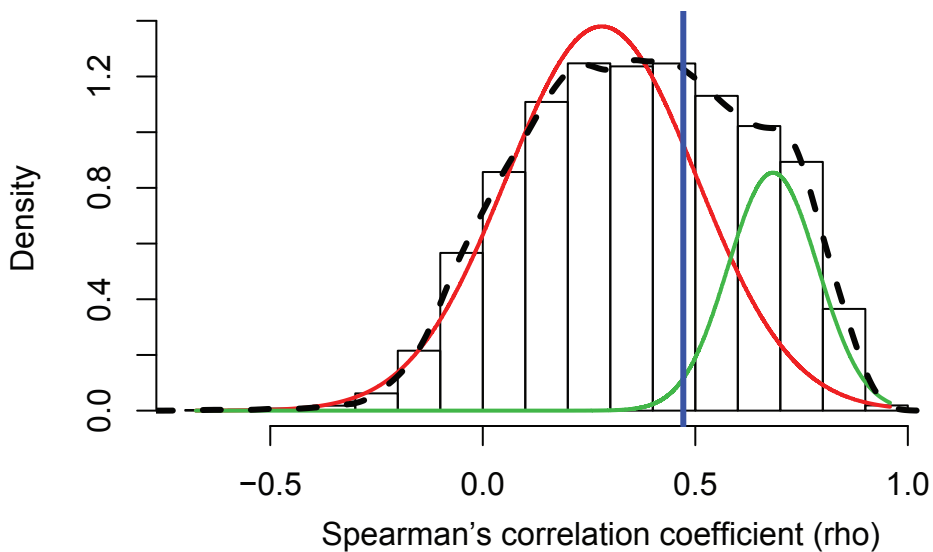**B**

### C3ARE Density Curves

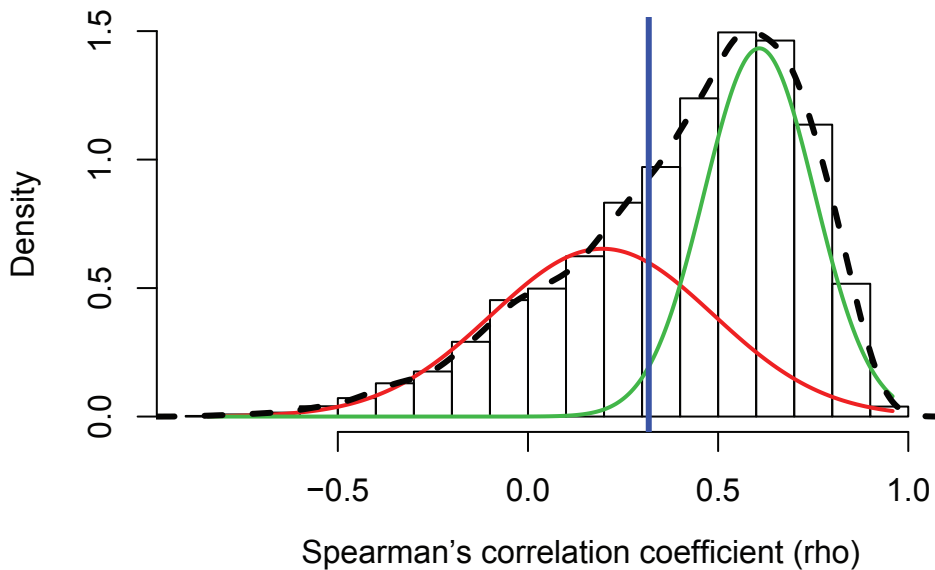

Supplement: Supplementary file 10 — Additional file 10: Fig. S3. Beta mixture modeling on Spearman correlation rho values between matched BECs and PBMCs for A) GECKO and b) C3ARE cohorts. The bimodal distribution of Spearman rho values indicated two underlying populations of CpGs, a set of uncorrelated CpGs (shown in red) and a set of right-skewed highly positively correlated CpGs (shown in green). Correlation coefficient threshold for informative CpGs were determined at two standard deviations minus the mean of the green Gaussian distribution (GECKO rho = 0.47; C3ARE rho = 0.32). [file 13072_2018_245_MOESM10_ESM.pdf]

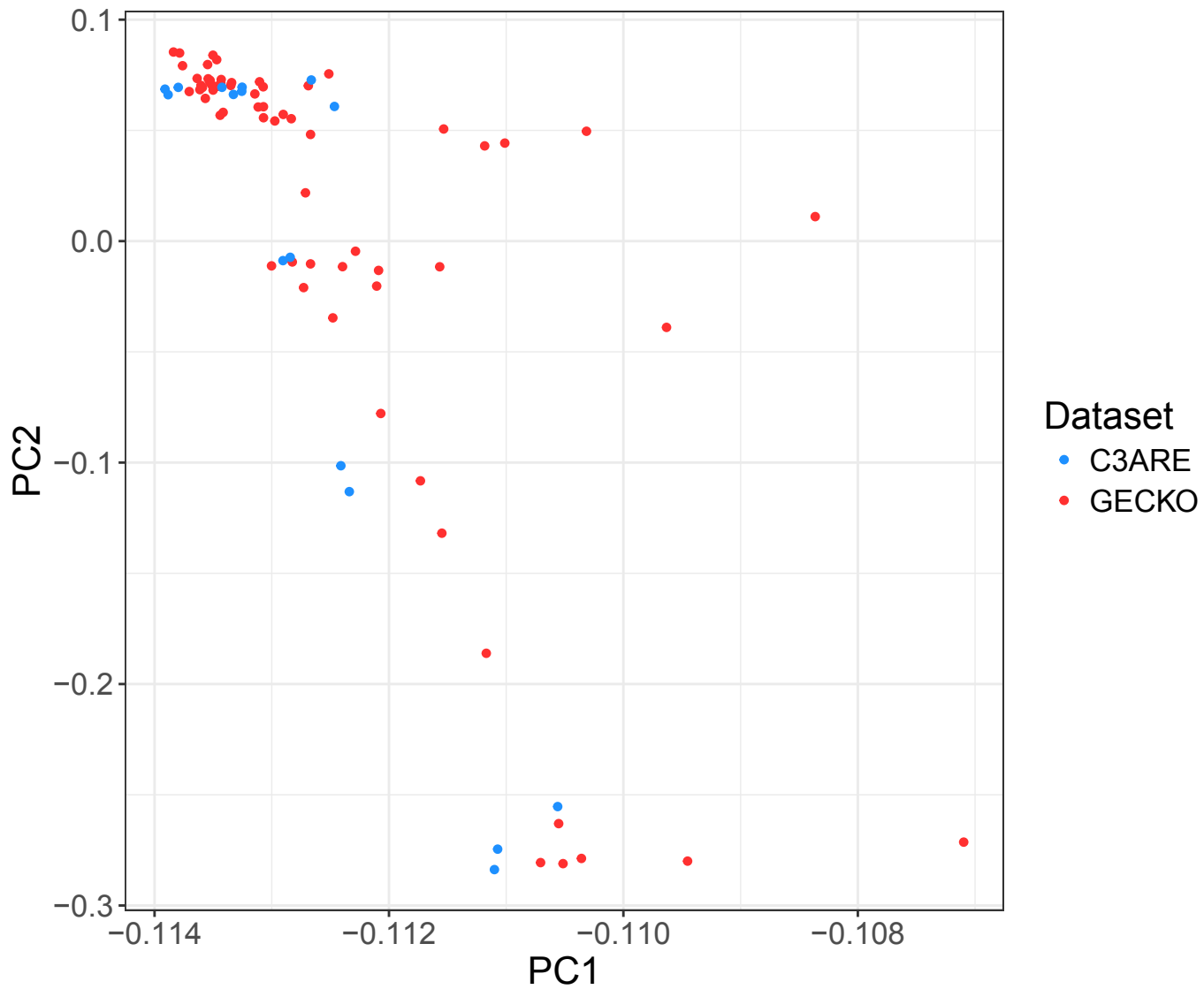

Supplement: Supplementary file 11 — Additional file 11: Fig. S4. Principal component analysis of PsychChip genotyping profiles (542,699 SNPs) for C3ARE (shown in blue) and GECKO (shown in red) revealed that genetic ancestry did not differ significantly between the cohorts as determined by Wilcoxon ranked-sum test of GECKO versus C3ARE in PC1 scores (p = 0.8) and PC2 scores (p = 0.4). [file 13072_2018_245_MOESM11_ESM.pdf]
