# Supplementary material for: Integration of DNA methylation patterns and genetic variation in human pediatric tissues help inform EWAS design and interpretation
Source: Epigenetics Chromatin. 2019 Jan 2;12:1. doi: 10.1186/s13072-018-0245-6 (PMC6314079; doi:10.1186/s13072-018-0245-6)
Supplement: Supplementary file 2 — Additional file 2: Table S1. The number of CpG sites at various thresholds of Spearman’s correlation rho and reference range for C3ARE, GECKO and GECKOsub datasets. [file 13072_2018_245_MOESM2_ESM.pdf]

Supplementary Table 1.

C3ARE		PBMC Reference Range				
		**0**	**≥ 0.05**	**≥ 0.1**	**≥ 0.2**	**≥ 0.5**
	**0**	419,507	64,204	12,218	1,742	46
**Positive**	**≥ 0.3**	74,137	24,542	7,474	1,282	44
**Correlation**	**≥ 0.6**	10,476	6,489	3,112	747	29
**Rho**	**≥ 0.9**	45	45	35	9	0
**Negative**	**≤ -0.3**	51,436	4,866	372	40	0
**Correlation**	**≤ -0.6**	3,300	327	34	6	0
**Rho**	**≤ -0.9**	3	0	0	0	0
**GECKO**		**PBMC Reference Range**				
		**0**	**≥ 0.05**	**≥ 0.1**	**≥ 0.2**	**≥ 0.5**
	**0**	419,507	131,227	28,311	3,597	159
**Positive**	**≥ 0.3**	333,22	29,158	15,774	3,055	146
**Correlation**	**≥ 0.6**	6,285	6,174	5,355	1,985	119
**Rho**	**≥ 0.9**	41	41	41	38	4
**Negative**	**≤ -0.3**	1,557	331	82	7	0
**Correlation**	**≤ -0.6**	8	8	6	0	0
**Rho**	**≤ -0.9**	0	0	0	0	0
**GECKOsub**		**PBMC Reference Range**				
		**0**	**≥ 0.05**	**≥ 0.1**	**≥ 0.2**	**≥ 0.5**
	**0**	419,507	115,404	21,563	2,689	93
**Positive**	**≥ 0.3**	30,615	26,385	12,916	2,306	88
**Correlation**	**≥ 0.6**	5,252	5,172	4,381	1,476	76
**Rho**	**≥ 0.9**	11	11	11	10	0
**Negative**	**≤ -0.3**	1,357	243	53	5	0
**Correlation**	**≤ -0.6**	5	5	4	0	0
**Rho**	**≤ -0.9**	0	0	0	0	0

The number of CpG sites at various thresholds of Spearman's correlation rho and reference range for C3ARE, GECKO and GECKOsub datasets.
