# Supplementary material for: Integration of DNA methylation patterns and genetic variation in human pediatric tissues help inform EWAS design and interpretation
Source: Epigenetics Chromatin. 2019 Jan 2;12:1. doi: 10.1186/s13072-018-0245-6 (PMC6314079; doi:10.1186/s13072-018-0245-6)
Supplement: Supplementary file 6 — Additional file 6: Table S2. The number of CpG sites of each defined category represented in reported significant hits of various pediatric EWAS publications. [file 13072_2018_245_MOESM6_ESM.pdf]

Supplementary Table 2.

Type of Site	# of Sites in Almstrup et al. 2017 (%)	# of Sites in Berko et al. 2014 (%)	# of Sites in Fisher et al. 2015 (%)	# of Sites in Portales et al. 2016 (%)	# of Sites in Xu et al. 2017 (%)
**All categories**	0 (0%)	0 (0%)	1 (0.4%)	3 (0.5%)	33 (0.3%)
**Differential**	67 (71.3%)	27 (36.5%)	42 (16.7%)	347 (52.7%)	6629 (68.3%)
**Informative**	1 (1.1%)	18 (24.3%)	2 (0.8%)	9 (1.4%)	63 (0.6%)
**Informative & Differential**	4 (0.04%)	6 (8.1%)	2 (0.8%)	21 (3.2%)	166 (1.7%)
**mQTL CpG**	2 (0.02%)	0 (0%)	0 (0%)	6 (0.9%)	45 (0.5%)
**mQTL & Differential**	0 (0%)	10 (13.5%)	2 (0.8%)	18 (2.7%)	168 (1.7%)
**mQTL & Informative**	0 (0%)	0 (0%)	1 (0.4%)	2 (0.3%)	9 (0.09%)
**None**	20 (21.3%)	13 (17.6%)	202 (80.2%)	252 (0.4%)	2591 (26.7%)
**Total Reported**	**94 (100%)**	**74 (100%)**	**252 (100%)**	**658 (100%)**	**9704 (100%)**

The number of CpG sites of each defined category represented in reported significant hits of various pediatric EWAS publications
